# Targeting macrophage polarization by inhibiting Pim2 alleviates inflammatory arthritis via metabolic reprogramming

**DOI:** 10.1038/s41423-025-01268-9

**Published:** 2025-02-26

**Authors:** Xiaojun Xu, Peitao Xu, Guozhen Shen, Xiaoshuai Peng, Zhidong Liu, Chaoqiang Chen, Wenhui Yu, Zepeng Su, Jiajie Lin, Guan Zheng, Guiwen Ye, Peng Wang, Zhongyu Xie, Yanfeng Wu, Huiyong Shen, Jinteng Li

**Affiliations:** 1 Department of Orthopedics, The Eighth Affiliated Hospital of Sun Yat-Sen University, 3025# Shennan Road, Shenzhen, 518033 PR China; 2 Guangdong Provincial Clinical Research Center for Orthopedic Diseases, The Eighth Affiliated Hospital, Sun Yat-sen University, 3025# Shennan Road, Shenzhen, 518033 PR China; 3 Center for Biotherapy, The Eighth Affiliated Hospital of Sun Yat-Sen University, 3025# Shennan Road, Shenzhen, 518033 PR China

**Keywords:** Inflammatory arthritis, Macrophage polarization, Glycolysis, Pim2, Bexarotene, Neutrophil membrane, Autoimmunity, Monocytes and macrophages, Drug delivery, High-throughput screening

## Abstract

Macrophage polarization and energy metabolic reprogramming play pivotal roles in the onset and progression of inflammatory arthritis. Moreover, although previous studies have reported that the proviral integration of Moloney virus 2 (Pim2) kinase is involved in various cancers through the mediation of aerobic glycolysis in cancer cells, its role in inflammatory arthritis remains unclear. In this study, we demonstrated that multiple metabolic enzymes are activated upon Pim2 upregulation during M1 macrophage polarization. Specifically, Pim2 directly phosphorylates PGK1-S203, PDHA1-S300, and PFKFB2-S466, thereby promoting glycolytic reprogramming. Pim2 expression was elevated in macrophages from patients with inflammatory arthritis and collagen-induced arthritis (CIA) model mice. Conditional knockout of Pim2 in macrophages or administration of the Pim2 inhibitor HJ-PI01 attenuated arthritis development by inhibiting M1 macrophage polarization. Through molecular docking and dynamic simulation, bexarotene was identified as an inhibitor of Pim2 that inhibits glycolysis and downstream M1 macrophage polarization, thereby mitigating the progression of inflammatory arthritis. For targeted treatment, neutrophil membrane-coated bexarotene (Bex)-loaded PLGA-based nanoparticles (NM@NP-Bex) were developed to slow the progression of inflammatory arthritis by suppressing the polarization of M1 macrophages, and these nanoparticles (NPs) exhibited superior therapeutic effects with fewer side effects. Taken together, the results of our study demonstrated that targeting Pim2 inhibition could effectively alleviate inflammatory arthritis via glycolysis inhibition and reversal of the M1/M2 macrophage imbalance. NM@NPs loaded with bexarotene could represent a promising targeted strategy for the treatment of inflammatory arthritis.

## Introduction

Inflammatory arthritis comprises various types of chronic progressive arthritis characterized by inflammation in the bones and joints, including rheumatoid arthritis (RA), ankylosing spondylitis (AS), psoriatic arthritis (PsA), systematic lupus erythematosus (SLE)-related arthritis and pediatric arthritis [1–3]. Despite variations in pathogenesis, excessive proinflammatory M1 macrophage polarization and reduced M2 macrophage polarization are common factors that contribute to the aggravation and deterioration of these types of arthritis [4–6]. Therefore, elucidating the mechanisms underlying aberrant macrophage polarization will lead to improved treatments for inflammatory arthritis.

An important feature of macrophages is their remarkable plasticity and ability to undergo rapid changes in morphology and status in response to their microenvironment to perform a variety of functions. The metabolic switch from oxidative phosphorylation to glycolysis, even in an oxygen-rich environment, is a hallmark of activated M1 macrophages; this metabolic switch, known as the Warburg effect, has also been observed in cancer cells. The switch to aerobic glycolysis is crucial for maintaining the activation and function of M1 macrophages [7–9]. Inhibiting glycolysis could effectively shift macrophages from the proinflammatory M1 phenotype to the immunosuppressive M2 phenotype [10–13]. As mentioned above, targeting M1/M2 macrophage polarization has been suggested as a promising therapeutic strategy for treating inflammatory arthritis. Therefore, inhibiting glycolysis to prevent M1 macrophage polarization may be a promising therapeutic strategy for treating inflammatory arthritis.

Proviral integration of Moloney virus 2 (Pim2) kinase is a Ca2+/calmodulin-dependent serine/threonine kinase belonging to the Pim kinase family. Previous studies have focused primarily on the role of Pim2 in tumorigenesis, and few investigations have explored whether Pim2 could be a potential target for treating inflammatory diseases [14–16]. Pim2 has been reported to act as a key kinase for the activation of several glycolytic enzymes, such as PFKFB3/4, HK2 and PKM2 [17–20]. On the basis of this idea, we hypothesized that targeting Pim2 may be an effective approach for inhibiting glycolysis and subsequently reducing M1 macrophage polarization.

In this study, we elucidated that Pim2 regulated M1 macrophage polarization via metabolic reprogramming. Pim2-mediated phosphorylation of several metabolic enzymes significantly attenuated OXPHOS but enhanced glycolysis, thereby promoting M1 macrophage polarization. Additionally, Pim2 was aberrantly overexpressed in macrophages in inflammatory arthritis lesions. Pim2 inhibition or knockdown in macrophages reduced M1 polarization but promoted M2 polarization, reversing the M1/M2 macrophage imbalance in vitro. Moreover, this reversal of the M1/M2 macrophage imbalance through the conditional knockout of Pim2 in macrophages or via the administration of the Pim2 inhibitor HJ-PI01 significantly slowed the pathological progression of inflammatory arthritis in collagen-induced arthritis (CIA) model mice, suggesting that Pim2 is a potential therapeutic target for treating inflammatory arthritis. Through molecular docking and dynamic simulation studies, bexarotene, an FDA-approved drug, was shown to inhibit Pim2 activity and suppress M1 macrophage polarization, thereby hindering the progression of CIA. For targeted delivery, we developed bexarotene-loaded poly(lactic-co-glycolic acid) (PLGA) nanoparticles coated with neutrophil membranes (NM@NP-Bex). Notably, NM@NP-Bex had a greater therapeutic effect on the skewed M1/M2 macrophage ratio and the severity and progression of CIA in vivo than did NP-Bex or NM@NPs alone, with minimal side effects. In summary, this study demonstrated that Pim2 inhibition effectively alleviated inflammatory arthritis via the inhibition of glycolysis and the subsequent regulation of M1 macrophage polarization. NM@NPs loaded with bexarotene could represent a promising targeted strategy for the treatment of inflammatory arthritis.

## Results

### Pim2 promotes glycolytic reprogramming in macrophages

**Fig. 1:**
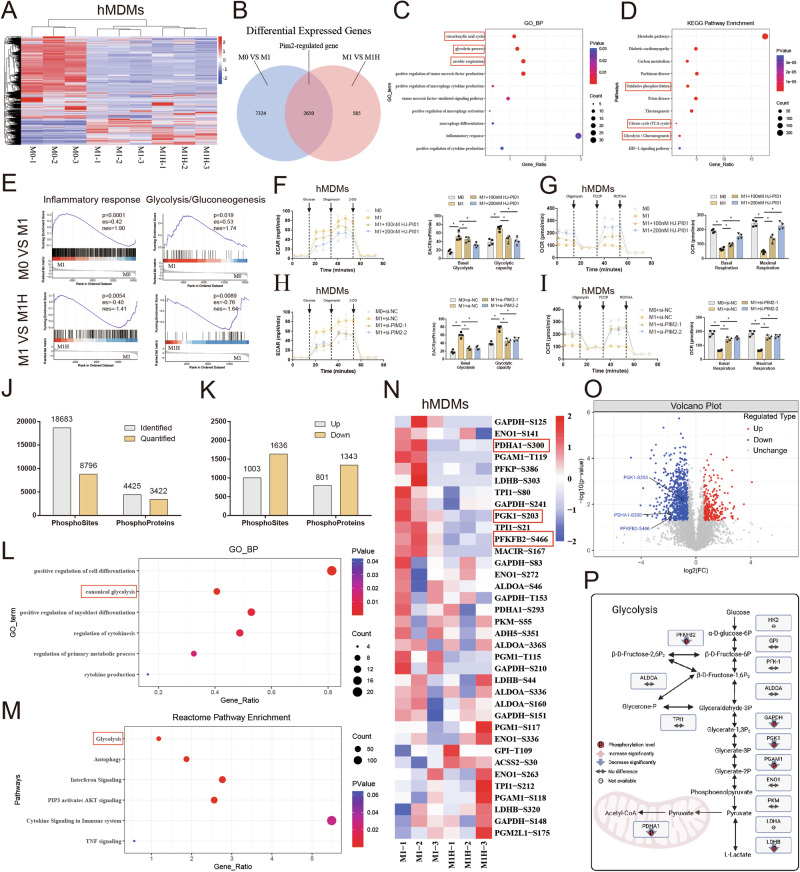
Pim2 promotes glycolytic reprogramming in macrophages. **A**, **B** Cluster heatmap and Venn plot of DEGs identified by RNA-seq in the M0, M1, and M1H groups (*n* = 3). **C**–**E** GO-BP, KEGG, and GSEA analyses of Pim2-regulated genes. **F** ECAR of hMDMs treated with or without HJ-PI01 after M1 induction (100 or 200 nM) (*n* = 4). **G** OCRs of hMDMs treated with or without HJ-PI01 (100 or 200 nM) after M1 induction (*n* = 4). **H** ECAR of hMDMs in the presence or absence of Pim2 knockdown after M1 induction (*n* = 4). **I** OCRs of hMDMs in the presence or absence of Pim2 knockdown after M1 induction (*n* = 4). **J** Histogram showing the number of phosphorylation sites and the corresponding proteins detected in hMDMs treated with or without 200 nM HJ-PI01 after M1 induction. **K** Histogram showing the number of Pim2-regulated phosphorylation sites and the corresponding proteins. **L**‒**M** GO-BP and Reactome pathway enrichment analyses of Pim2-regulated proteins. **N** Cluster heatmap of metabolic enzymes (*n* = 3). **O** Volcano plots of metabolic enzymes identified by phosphoproteomic analysis of the M1 and M1H groups. **P** Changes in the protein and phosphorylation levels of metabolic enzymes

To elucidate the role of Pim2 in macrophages, circulating monocytes were isolated from healthy donors and then further differentiated into macrophages (hMDMs) in vitro, followed by M1 induction with lipopolysaccharide (LPS)/interferon-γ (IFN-γ) and Pim2 inhibition with HJ-PI01. Then, RNA-seq was conducted using untreated hMDMs (M0 group), LPS/IFN-γ-treated hMDMs (M1 group) and LPS/IFN-γ- and HJ-PI01-treated hMDMs (M1H group). The results revealed 9974 DEGs between the M0 and M1 groups and 3235 DEGs between the M1 and M1H groups. Moreover, 2650 key DEGs were regulated by Pim2, as visualized in the Venn diagram (Fig. 1A, B). Gene Ontology (GO) analysis revealed that biological processes such as glycolysis, positive regulation of macrophage cytokine production, positive regulation of macrophage activation, and macrophage differentiation signaling pathways were enriched in these key genes (Fig. 1C). Kyoto Encyclopedia of Genes and Genomes (KEGG) pathway analysis further revealed that “glycolysis/gluconeogenesis,” “oxidative phosphorylation,” and the “tricarboxylic acid cycle” were enriched predominantly among the Pim2-regulated genes (Fig. 1D). Additionally, gene set enrichment analysis (GSEA) revealed that inflammatory response- and glycolysis-related pathways were enriched in the upregulated genes between the M0 and M1 groups. Moreover, the genes whose expression was downregulated between the M1H and M1 groups were enriched in these pathways. Subsequent qPCR also validated the expression of inflammation-related genes and glycolysis-related genes, which roughly aligned with the results from transcriptome sequencing (Supplementary Fig. S1A–C). These findings suggest that Pim2 may regulate the metabolic reprogramming of macrophages.

Macrophage polarization is intricately linked to cellular metabolism and changes in metabolic signaling pathways. M1 macrophages rely on glycolysis, whereas M2 macrophages utilize oxidative phosphorylation [7–9]. To elucidate the role of Pim2 in the metabolic reprogramming of macrophages, we assessed glycolysis and oxidative phosphorylation via the extracellular acidification rate (ECAR) and oxygen consumption rate (OCR). Compared with untreated hMDMs, LPS/IFN-γ-treated hMDMs presented a greater ECAR and a lower OCR; however, HJ-PI01 treatment reversed these effects (Fig. 1F, G). Additionally, compared with untreated hMDMs, LPS/IFN-γ-treated hMDMs presented elevated lactate levels and reduced ATP levels, and these effects were reversed by HJ-PI01 treatment (Supplementary Fig. S3A, B). These findings suggest that Pim2 promotes glycolytic reprogramming in macrophages.

To further elucidate the role of Pim2 in metabolic reprogramming in macrophages, we designed three specific siRNAs targeting Pim2. siRNA1 and siRNA2 significantly reduced Pim2 expression in hMDMs (Supplementary Fig. S2A, B). Compared with those treated with si-NC, LPS/IFN-γ-treated hMDMs treated with si-Pim2-1 or si-Pim2-2 exhibited a decreased ECAR and an increased OCR (Fig. 1H, I). Additionally, Pim2 knockdown reduced the level of lactate and increased the level of ATP in LPS/IFN-γ-treated hMDMs, indicating that Pim2 knockdown promotes the transition from glycolysis to oxidative phosphorylation in macrophages (Supplementary Fig. S3C, D). These findings further suggest that Pim2 promotes glycolytic reprogramming in macrophages.

Protein phosphorylation plays a crucial role in regulating glycolysis and oxidative phosphorylation [21]. Pim2 modulates glycolytic activity by mediating the phosphorylation of various glycolytic enzymes [17–20]. To further elucidate the specific mechanisms by which Pim2 regulates metabolic reprogramming in macrophages, we analyzed the protein phosphorylation profiles of LPS/IFN-γ-treated hMDMs (M1 group) and LPS/IFN-γ- and HJ-PI01-treated hMDMs (M1H group). We identified a total of 18,683 phosphorylation sites in 4,425 proteins, 8,796 of which were quantifiable (Fig. 1J). Compared with those in the M1 group, the phosphorylation levels of 801 proteins (1,003 sites) were increased, whereas the phosphorylation levels of 1,343 proteins (1,636 sites) were decreased in the M1H group (Fig. 1K). GO-BP and Reactome pathway enrichment analyses of proteins with decreased phosphorylation levels revealed significant enrichment in the glycolytic pathway (Fig. 1L, M). These results indicate that Pim2 potentially regulates the metabolic reprogramming of macrophages via metabolic enzyme phosphorylation.

### Pim2 promotes glycolytic reprogramming via metabolic enzyme phosphorylation

**Fig. 2:**
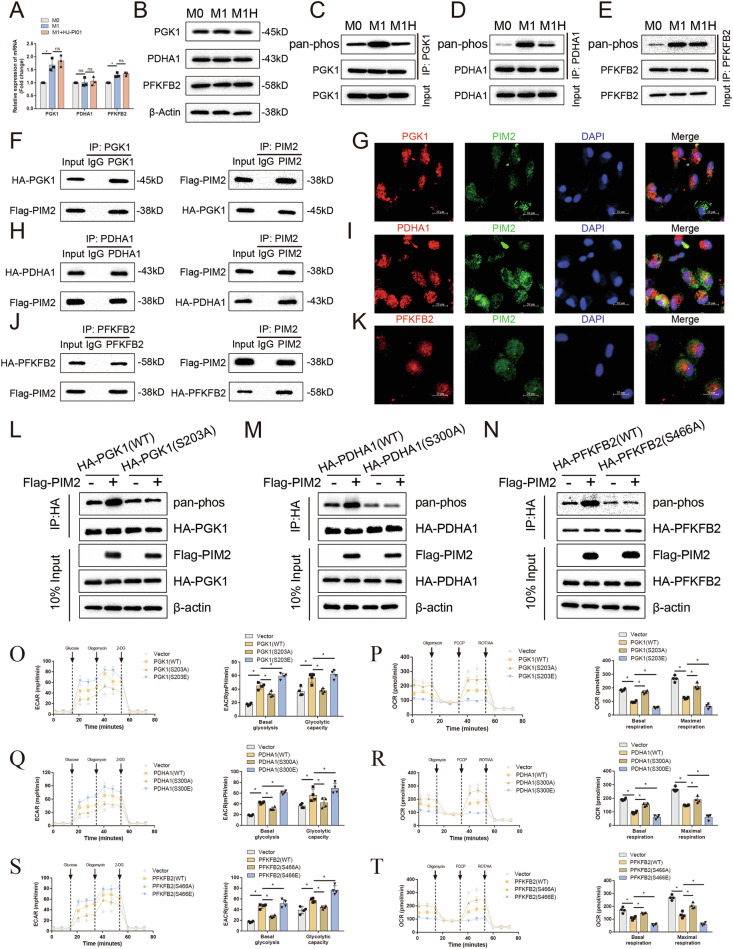
Pim2 regulates glycolytic activity via phosphorylation of glycolytic enzymes. **A**, **B** The mRNA and protein levels of PGK1, PDHA1, and PFKFB2 in hMDMs with or without 200 nM HJ-PI01 treatment after M1 induction were determined by RT‒qPCR and Western blotting (*n* = 3). **C**–**E** The phosphorylation levels of PGK1, PDHA1, and PFKFB2 in hMDMs with or without 200 nM HJ-PI01 treatment after M1 induction were determined by immunoprecipitation followed by Western blotting (*n* = 3). HEK293T cells were transfected with the indicated HA-tagged PGK1 (**F**‒**G**), PDHA1 (**H**‒**I**), and PFKFB2 (**J**‒**K**), along with Flag-tagged Pim2 proteins. Immunoprecipitation and immunofluorescence staining were performed using anti-HA and anti-Flag antibodies. **L-N** HEK293T cells were transfected with the indicated HA-tagged PGK1 (WT or S203A), PDHA1 (WT or S300A), and PFKFB2 (WT or S466A), along with Flag-tagged Pim2 proteins. The phosphorylation levels of PGK1, PDHA1, and PFKFB2 in HEK293T cells were determined by immunoprecipitation followed by Western blotting (*n* = 3). **O**–**T** PGK1 (WT, S203A, or S203E), PDHA1 (WT, S300A, or S300E), and PFKFB2 (WT, S466A, or S466E) were overexpressed in HEK293T cells. The ECAR and OCR of the different groups of HEK293T cells were determined

To investigate the specific mechanism by which Pim2 regulates the metabolic reprogramming of macrophages, we analyzed the phosphorylation levels of metabolic enzymes in macrophages via phosphoproteomic data. The analyses revealed that HJ-PI01 treatment reduced the phosphorylation levels of several metabolic enzymes, including PGK1-S203, PDHA1-S300, PFKFB2-S466, ADH5-S351, GAPDH-S210, GAPDH-S125, LDHB-S203 and PGAM1-T119, in LPS/IFN-γ-treated hMDMs, as demonstrated in the heatmap and volcano plot in Fig. 1N‒P. We first examined the expression levels of these metabolic enzymes and found that HJ-PI01 treatment did not alter their mRNA or protein levels (Fig. S4A, B). Further examination of the phosphorylation levels of these enzymes revealed that the phosphorylation levels of PGK1, PDHA1, and PFKFB2 were greater in the LPS/IFN-γ-treated hMDMs than in the untreated hMDMs but decreased after HJ-PI01 treatment (Fig. 2C–E). In contrast, the phosphorylation levels of the other proteins were not significantly altered (Supplementary Fig. S4C–F). These results suggest that Pim2 may regulate M1 macrophage polarization by modulating the phosphorylation levels of PGK1, PDHA1 and PFKFB2.

To verify the interaction between Pim2 and the metabolic enzymes PGK1, PDHA1, and PFKFB2, we exogenously expressed Flag-Pim2 in combination with HA-tagged PGK1, PDHA1, and PFKFB2 in 293T cells. Coimmunoprecipitation (co-IP) assays demonstrated that Pim2 binds to PGK1, PDHA1, and PFKFB2 (Fig. 2F, H, J). Subsequent confocal microscopy analysis of 293T cells revealed that Pim2 and these metabolic enzymes colocalized (Fig. 2G, I, K). Given the localization of the PDHA1 protein in the mitochondrial matrix, the cellular localization of Pim2 was investigated. We confirmed the presence of Pim2 in both the cytoplasm and the mitochondrial matrix via mitochondrial fractionation, Western blotting and immunofluorescence staining (Supplementary Fig. S5A, B). These findings confirm the interaction between Pim2 and the metabolic enzymes PGK1, PDHA1, and PFKFB2.

To pinpoint the specific sites where Pim2 regulates PGK1, PDHA1, and PFKFB2, we next constructed wild-type and phosphorylation-inactive point mutant proteins, PGK1 (WT or S203A), PDHA1 (WT or S300A), and PFKFB2 (WT or S466A), and overexpressed them alongside Pim2 in 293T cells. As shown in Fig. 2L, the S203A mutation in PGK1 blocked Pim2-mediated phosphorylation compared with that in wild-type PGK1, indicating that S203 is a critical site for Pim2 regulation of PGK1 (Fig. 2L). Similarly, the S300A mutation in PDHA1 blocked Pim2-mediated phosphorylation relative to that in wild-type PDHA1, suggesting that S300 is a key site for Pim2 regulation of PDHA1 (Fig. 2M). Compared with wild-type PFKFB2, the S466A mutation blocked Pim2-mediated phosphorylation, indicating that S466 is a crucial site for Pim2 regulation of PFKFB2 (Fig. 2N).

To further investigate the impact of Pim2-mediated phosphorylation on metabolic enzyme activity, we constructed wild-type, phosphorylation-inactive and phosphorylation-active point mutant proteins, PGK1 (WT, S203A, or S203E), PDHA1 (WT, S300A, or S300E), and PFKFB2 (WT, S466A, or S466E), and overexpressed them in 293T cells. Compared with PGK1-WT, the S203A mutation in PGK1 decreased the ECAR and increased the OCR of 293T cells, whereas the S203E mutation had the opposite effect (Fig. 2O–P). Similarly, compared with PDHA1-WT, the S300A mutation in PDHA1 inhibited the ECAR and increased the OCR, whereas the S300E mutation had the opposite effect (Fig. 2Q, R). In the case of PFKFB2, the S466A mutation inhibited the ECAR and increased the OCR compared with those of PFKFB2-WT, whereas the S466E mutation had the opposite effect (Fig. 2S, T). These data indicate that the phosphorylation of PGK1 at S203, PDHA1 at S300, and PFKFB2 at S466 plays crucial roles in Pim2-mediated metabolic reprogramming.

### Macrophage Pim2 expression is elevated in patients with inflammatory arthritis and in CIA model mice

**Fig. 3:**
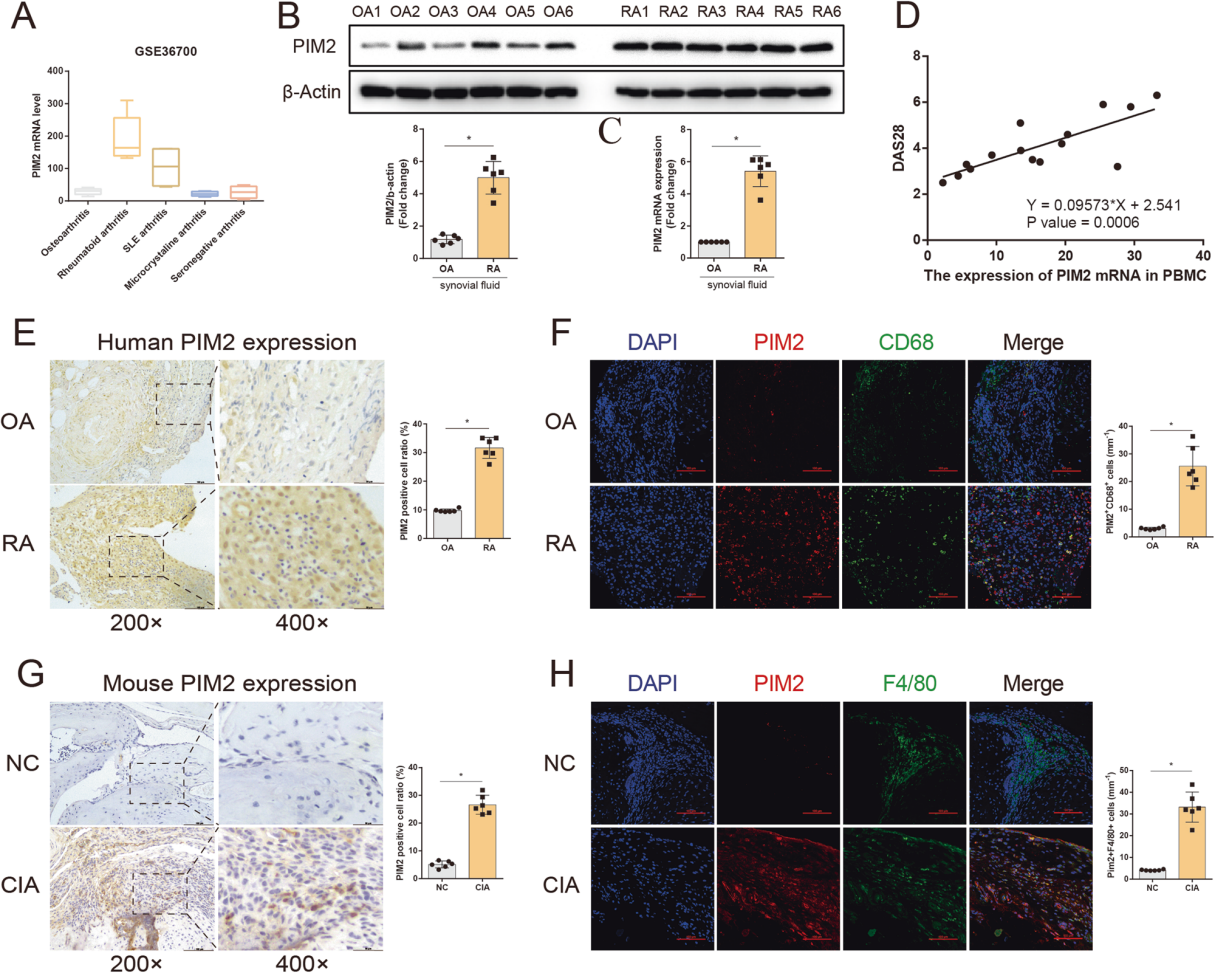
Macrophage Pim2 expression is elevated in patients with inflammatory arthritis and in CIA model mice. **A** The expression of Pim2 in the synovial tissue of patients with arthritis was obtained from the public dataset GEO36700. **B**, **C** The mRNA and protein levels of Pim2 in the synovial tissues of patients with OA and RA (*n* = 6). **D** Pim2 was upregulated in the PBMCs of RA patients and correlated with DAS28. **E** Representative IHC images showing Pim2 expression in the synovium of OA and RA patients (*n* = 6). **F** Representative immunofluorescence images showing Pim2 expression in CD68+ macrophages in the synovial tissues of OA and RA patients (CD68, green; Pim2, red) (*n* = 6). **G** Representative IHC images showing Pim2 expression in the synovial tissues of NC and CIA model mice (*n* = 6). **H** Representative immunofluorescence images showing Pim2 expression in F4/80+ macrophages in the synovial tissues of NC and CIA model mice (F4/80, green; Pim2, red) (*n* = 6)

To investigate the relationship between inflammatory arthritis and Pim2 expression, we analyzed public data (GEO36700) to determine the mRNA levels of Pim2 in patients with various forms of arthritis, including serum-negative arthritis, microcrystalline arthritis, SLE-related arthritis, osteoarthritis (OA), and rheumatoid arthritis (RA). Pim2 was found to be more highly expressed in patients with RA than in patients with other types of arthritis (Fig. 3A). To further verify the expression of Pim2 in synovial tissues, we examined the knee joint synovial tissues of OA and RA patients. Compared with those in the synovial tissue of the OA group, the mRNA and protein expression levels of Pim2 were significantly greater in the synovial tissue of the RA group (Fig. 3B, C). We further examined the diagnostic value of Pim2 in the PBMCs of RA patients and found that the mRNA level of Pim2 in PBMCs was positively correlated with the disease activity score (DAS28), which reflects the severity of RA (Fig. 3D).

Immunohistochemical (IHC) analysis revealed that Pim2 expression was significantly higher in the RA group than in the OA group (Fig. 3E). Compared with that in the OA group, the infiltration of CD68+ macrophages in the synovial tissue of RA patients was markedly greater, and the expression of Pim2 in these CD68+ macrophages was significantly greater (Fig. 3F). In the CIA mouse model, a classic animal model for studying arthritis, chronic synovial inflammation and M1 macrophage polarization are crucial for the development and maintenance of arthritis [22]. Therefore, we also measured Pim2 expression in ankle joint tissue from CIA model mice and normal control (NC) mice. IHC results demonstrated that Pim2 expression was significantly increased in the CIA group (Fig. 3G). The immunofluorescence results indicated that the infiltration of F4/80+ macrophages in the synovial tissue of CIA model mice was significantly greater than that in control mice and that Pim2 expression in these F4/80+ macrophages was markedly increased (Fig. 3H). These findings suggest that Pim2 expression is elevated in macrophages in inflammatory arthritis.

### Pim2 promotes M1 macrophage polarization in vitro

**Fig. 4:**
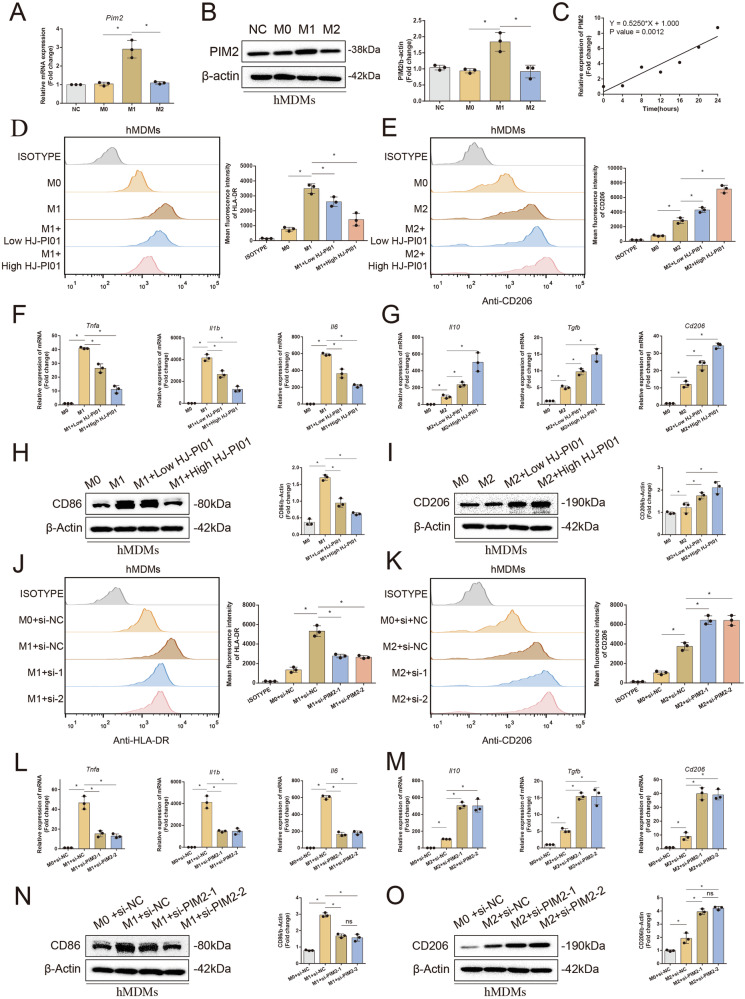
Pim2 promotes M1 macrophage polarization in vitro. **A**, **B** The mRNA and protein levels of Pim2 during M1/M2 polarization of hMDMs were determined by qRT‒PCR and Western blotting (*n* = 3). **C** Pim2 expression was positively correlated with M1 polarization. **D**, **E** MFI of HLA-DR and CD206 in CD68+ hMDMs treated with or without HJ-PI01 (100 or 200 nM) after M1/M2 induction, as detected by flow cytometry (*n* = 3). **F**–**I** mRNA and protein expression levels of inflammatory factors in hMDMs treated with or without HJ-PI01 (100 or 200 nM) after M1/M2 induction, as determined by RT‒qPCR and Western blotting (*n* = 3). **J**–**K** MFI of HLA-DR and CD206 in CD68+ hMDMs in the presence or absence of Pim2 knockdown after M1/M2 induction, as detected by flow cytometry (*n* = 3). **L**‒**O** mRNA and protein expression levels of inflammatory factors in hMDMs in the presence or absence of Pim2 knockdown after M1/M2 induction, as determined by RT‒qPCR and Western blotting (*n* = 3)

Correcting the skewed M1/M2 macrophage ratio in the joints of RA patients could mitigate joint damage, as M1 macrophages appear to be able to drive the pathological process of RA [23]. To further investigate the function of Pim2 in macrophage polarization, we used LPS/IFN-γ and IL-4 to induce M1-like and M2-like macrophages, respectively, in vitro. M1 induction significantly increased Pim2 mRNA and protein levels, but M2 induction did not change Pim2 mRNA or protein levels in hMDMs (Fig. 4A–C), suggesting that Pim2 plays a critical role in the regulation of M1 macrophage polarization.

To investigate the effect of Pim2 inhibition on M1 or M2 macrophage polarization, we measured the expression of an M1 marker (HLA-DR) and an M2 marker (CD206) in HJ-PI01-treated hMDMs via flow cytometry. Inhibiting Pim2 with HJ-PI01 significantly decreased the mean fluorescence intensity (MFI) of HLA-DR in hMDMs after M1 induction (Fig. 4D). Conversely, HJ-PI01 treatment increased the MFI of CD206 in hMDMs after M2 induction (Fig. 4E). M1 macrophages are known to express proinflammatory cytokines, such as IL-1β, IL-6, and TNF-α, whereas M2 macrophages express anti-inflammatory cytokines, such as IL-10, TGF-β and CD206 [24]. HJ-PI01 significantly decreased the mRNA levels of M1-associated genes (IL-1β, IL-6, and TNF-α) in hMDMs after M1 induction but increased the mRNA levels of M2-mediated genes (IL-10, TGF-β, and CD206) in hMDMs after M2 induction (Fig. 4F, G). Consistent with these findings, Western blot analysis revealed that HJ-PI01 reduced the protein level of CD86 in hMDMs after M1 induction but increased the protein level of CD206 in hMDMs after M2 induction (Fig. 4H, I). These results reveal that Pim2 inhibition reduces M1 polarization but enhances M2 polarization in macrophages.

To further investigate the effect of Pim2 knockdown on M1 or M2 macrophage polarization, we measured M1 and M2 markers in hMDMs treated with si-Pim2 via flow cytometry. Pim2 knockdown decreased the MFI of HLA-DR in hMDMs after M1 induction but increased the MFI of CD206 in hMDMs after M2 induction (Fig. 4J, K). Pim2 knockdown also decreased the mRNA levels of IL-1β, IL-6, and TNF-α in hMDMs after M1 induction but increased the mRNA levels of IL-10, TGF-β and CD206 in hMDMs after M2 induction (Fig. 4L, M). Consistent with these results, Western blot analysis revealed that Pim2 knockdown reduced the protein level of CD86 in hMDMs after M1 induction and increased the protein level of CD206 in hMDMs after M2 induction (Fig. 4N, O). These observations indicate that Pim2 knockdown reduces M1 polarization but enhances M2 polarization in macrophages.

These results suggest that Pim2 promotes the proinflammatory response of macrophages by influencing M1/M2 differentiation.

### Pim2 expression in macrophages is involved in the development of inflammatory arthritis

To explore the role of Pim2 in inflammatory arthritis, we generated macrophage-specific Pim2 knockout mice (Pim2^fl/fl^-Lyz2^Cre^ mice) and their littermate controls (Pim2^fl/fl^ mice) and subjected them to arthritis induction (Supplementary Fig. S6A). All the mice were genotyped via DNA electrophoresis (Supplementary Fig. S6D). The absence of Pim2 in bone marrow-derived macrophages (BMDMs) from Pim2^fl/fl^-Lyz2^Cre^ mice was confirmed by Western blotting and RT‒qPCR (Supplementary Fig. S6B, C).

**Fig. 5:**
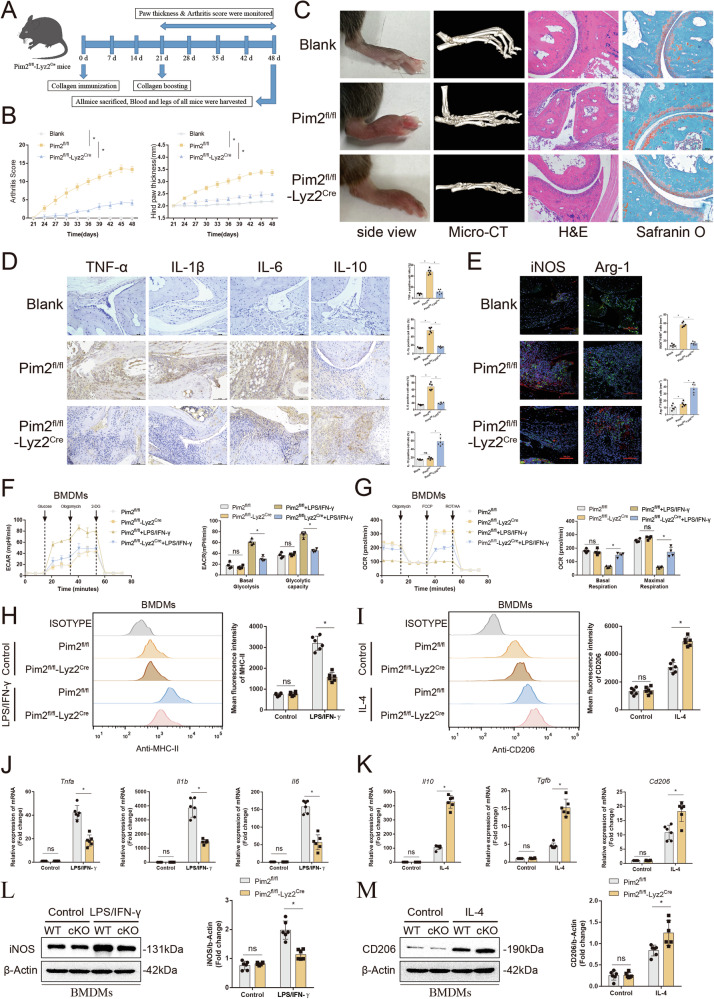
Pim2 expression in macrophages is involved in the development of inflammatory arthritis. **A** Schematic of CIA induction in Pim2^fl/fl^-Lyz2^Cre^ mice. **B** CIA scores and hind paw thicknesses of Pim2^fl/fl^ and Pim2^fl/fl^-Lyz2^Cre^ mice (*n* = 6). **C** Macroscopic images, micro-CT, H&E, and safranin O staining of the ankles of Pim2^fl/fl^ and Pim2^fl/fl^-Lyz2^Cre^ mice (*n* = 6). **D** The expression of TNF-α, IL-1β, IL-6 and IL-10 in Pim2^fl/fl^ and Pim2^fl/fl^-Lyz2^Cre^ mice after CIA induction, as detected by IHC. **E** The expression of iNOS and Arg-1 in synovial macrophages from Pim2^fl/fl^ and Pim2^fl/fl^-Lyz2^Cre^ mice, as detected by IF staining (F4/80, green; iNOS, red; Arg-1, red). **F**, **G** ECARs and OCRs of BMDMs isolated from Pim2^fl/fl^ and Pim2^fl/fl^-Lyz2^Cre^ mice after M1 induction (*n* = 4). **H**, **I** MFIs of MHC-II and CD206 in F4/80+ BMDMs isolated from Pim2^fl/fl^ and Pim2^fl/fl^-Lyz2^Cre^ mice after M1/M2 induction, as detected by flow cytometry (*n* = 6). **J**‒**M** mRNA and protein expression levels of inflammatory factors in BMDMs isolated from Pim2^fl/fl^ and Pim2^fl/fl^-Lyz2^Cre^ mice after M1/M2 induction, as determined by RT‒qPCR and Western blotting (*n* = 6)

The CIA score was lower, and the hind paw thickness was lower in Pim2^fl/fl^-Lyz2^Cre^ mice than in Pim2^fl/fl^ mice (Fig. 5B). Consistently, Pim2^fl/fl^ mice exhibited severe swelling, redness, and inflammatory infiltration in the joints following arthritis induction, and these effects were significantly ameliorated in Pim2^fl/fl^-Lyz2^Cre^ mice (Fig. 5C). Additionally, the severe cartilage and bone destruction observed in Pim2^fl/fl^-Lyz2^Cre^ mice was markedly reduced compared with that in Pim2^fl/fl^ mice (Fig. 5C). These findings indicate that Pim2 deficiency in macrophages mitigates the development of inflammatory arthritis.

The overproduction of inflammatory factors is recognized as a key regulator driving synovial inflammation and joint destruction. Therefore, we examined the levels of inflammatory cytokines in the synovial tissues of mice via IHC staining. As expected, compared with those in the blank group, the number of TNF-α-, IL-1β-, and IL-6-positive cells was significantly greater, whereas the number of IL-10-positive cells remained low in the Pim2^fl/fl^ group. However, in the Pim2^fl/fl^-Lyz2^Cre^ group, the number of TNF-α-, IL-1β-, and IL-6-positive cells was significantly decreased, whereas the number of IL-10-positive cells showed the opposite trend (Fig. 5D), suggesting that myeloid Pim2 deficiency reduces M1 polarization but enhances M2 polarization of macrophages in vivo. iNOS and Arg-1 are markers of M1-type and M2-type macrophages, respectively. We conducted immunofluorescence staining of synovial membranes from mice across all experimental groups, employing double staining for iNOS/Arg-1 and F4/80. As anticipated, the Pim2^fl/fl^ group presented a significant increase in the number of iNOS+F4/80 + M1 macrophages, whereas the number of Arg-1 + F4/80 + M2 macrophages remained markedly low. Conversely, in the Pim2^fl/fl^-Lyz2^Cre^ group, there was a notable reduction in the number of iNOS+F4/80 + M1 macrophages, accompanied by an increase in the number of Arg-1 + F4/80 + M2 macrophages (Fig. 5E). These findings further confirmed that myeloid Pim2 deficiency reduces M1 polarization but enhances M2 polarization in macrophages in vivo.

To elucidate the mechanism by which Pim2 in macrophages exacerbates inflammatory arthritis, we conducted a comparative analysis of glycolytic and oxidative phosphorylation activities in BMDMs isolated from Pim2^fl/fl^-Lyz2^Cre^ and Pim2^fl/fl^ mice, followed by treatment with LPS/IFN-γ. Moreover, the ECAR of LPS/IFN-γ-treated BMDMs from Pim2^fl/fl^-Lyz2^Cre^ mice was decreased, whereas their OCR was increased (Fig. 5F, G). These findings suggest that myeloid Pim2 deficiency facilitates the metabolic transition from glycolysis to oxidative phosphorylation in macrophages.

We next determined the M1/M2 polarization of BMDMs isolated from Pim2^fl/fl^-Lyz2^Cre^ and Pim2^fl/fl^ mice. Consistently, the MFI of MHC-II was significantly lower in BMDMs from Pim2^fl/fl^-Lyz2^Cre^ mice than in those from Pim2^fl/fl^ mice after M1 induction. Conversely, the MFI of CD206 was significantly greater in BMDMs from Pim2^fl/fl^-Lyz2^Cre^ mice than in those from Pim2^fl/fl^ mice (Fig. 5H, I). Similar results were observed for the expression of key inflammatory factors in M1/M2 macrophages. Compared with those from Pim2^fl/fl^-Lyz2^Cre^ mice, BMDMs from Pim2^fl/fl^-Lyz2^Cre^ mice expressed lower mRNA levels of TNF-α, IL-1β, and IL-6 after M1 induction (Fig. 5J). Conversely, BMDMs from Pim2^fl/fl^-Lyz2^Cre^ mice expressed higher mRNA levels of IL-10, TGF-β, and CD206 than did those from Pim2^fl/fl^ mice after M2 induction (Fig. 5K). Additionally, the protein levels of iNOS were significantly decreased after M1 induction, whereas the protein levels of CD206 were increased after M2 induction in Pim2^fl/fl^-Lyz2^Cre^ mice compared with Pim2^fl/fl^ mice (Fig. 5L, M). These results suggest that myeloid Pim2 deficiency reduces M1 polarization but enhances M2 polarization in macrophages in vitro.

These findings suggest that Pim2 exacerbates inflammatory arthritis primarily by promoting glycolysis and M1 polarization in macrophages.

### Pim2 is a therapeutic target for inflammatory arthritis

**Fig. 6:**
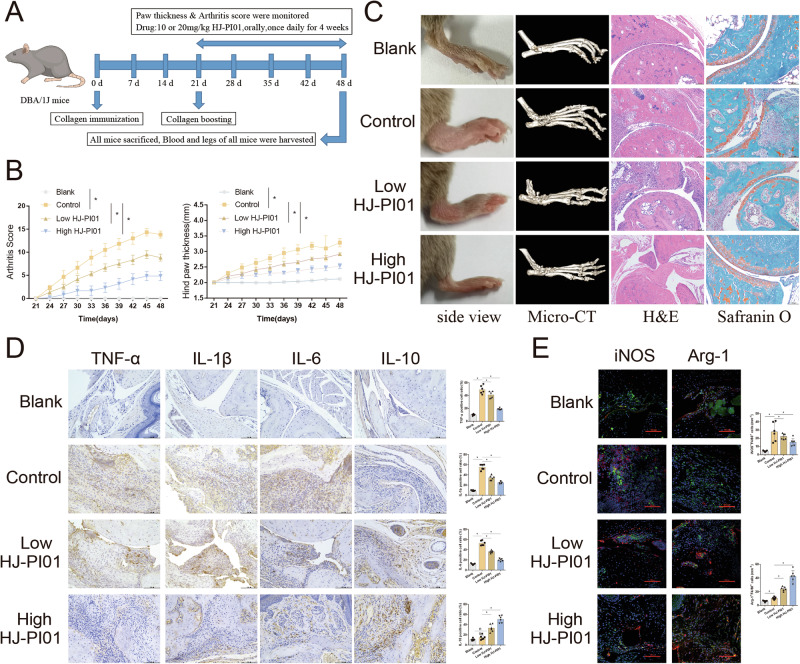
Pim2 is a therapeutic target for inflammatory arthritis. **A** Schematic of CIA model mice treated with HJ-PI01 (10 or 20 mg/kg). **B** CIA scores and hind paw thickness of the mice in each group (*n* = 6). **C** Macroscopic images, micro-CT, H&E, and safranin O staining of the ankles from the mice in each group (*n* = 6). **D** The expression of TNF-α, IL-1β, IL-6 and IL-10 in the mice in each group, as detected by IHC (*n* = 6). **E** The expression of iNOS and Arg-1 in the synovial macrophages of the mice in each group, as detected by immunofluorescence staining (F4/80, green; iNOS, red; Arg-1, red) (*n* = 6)

Given that Pim2 exacerbates inflammatory arthritis, as suggested by the findings above, inhibiting Pim2 may slow the progression of this disease. To test this hypothesis, CIA model mice were generated, and the Pim2 inhibitor HJ-PI01 was administered orally (Fig. 6A). The CIA score was lower, and the hind paw thickness was lower in the HJ-PI01 group than in the control group (Fig. 6B). Consistently, HJ-PI01 treatment alleviated swelling and redness, decreased inflammatory cell infiltration, and reduced cartilage erosion and bone destruction in CIA model mice, as demonstrated by H&E staining, safranin O staining, and micro-CT, respectively (Fig. 6C).

Next, we measured the levels of inflammatory cytokines in the synovial tissues of the mice via IHC staining. Compared with those in the control group, the number of TNF-α-, IL-6-, and IL-1β-positive cells in the HJ-PI01 group was significantly lower, whereas the number of IL-10-positive cells was markedly greater (Fig. 6D). Furthermore, immunofluorescence staining of the synovium revealed a substantial decrease in the number of iNOS+F4/80 + M1 macrophages and a significant increase in the number of Arg-1 + F4/80 + M2 macrophages in the HJ-PI01 group (Fig. 6E). These findings corroborate that Pim2 inhibition with HJ-PI01 reduces M1 polarization but enhances M2 polarization in macrophages in vivo.

To confirm that the immune modulation of HJ-PI01 is specifically mediated by the inhibition of PIM2, we conducted genetic knockout experiments in both cell and animal models. At the cellular level, HJ-PI01 treatment inhibited M1 polarization in BMDMs derived from Pim2^fl/fl^ mice while promoting M2 polarization. In contrast, BMDMs from Pim2^fl/fl^-Lyz2^Cre^ mice presented reduced M1 polarization and enhanced M2 polarization, and HJ-PI01 treatment did not further affect their M1/M2 polarization balance (Supplementary  Fig. S7A–F). At the animal level, HJ-PI01 treatment improved arthritis symptoms in Pim2^fl/fl^ mice by reducing M1 macrophage infiltration in synovial tissues and increasing M2 macrophage infiltration. However, in Pim2^fl/fl^-Lyz2^Cre^ mice, which exhibited only mild arthritis symptoms, HJ-PI01 treatment had no further effect on arthritis progression (Supplementary Fig. S7G–K). These results are sufficient to indicate that HJ-PI01 exerts its regulatory effect on macrophage polarization by targeting PIM2 inhibition, thereby improving inflammatory arthritis.

These results suggest that targeting Pim2 to inhibit M1 macrophage polarization may be a therapeutic strategy for treating inflammatory arthritis.

### The FDA-approved drug bexarotene inhibits macrophage M1 polarization and alleviates arthritis in CIA model mice

**Fig. 7:**
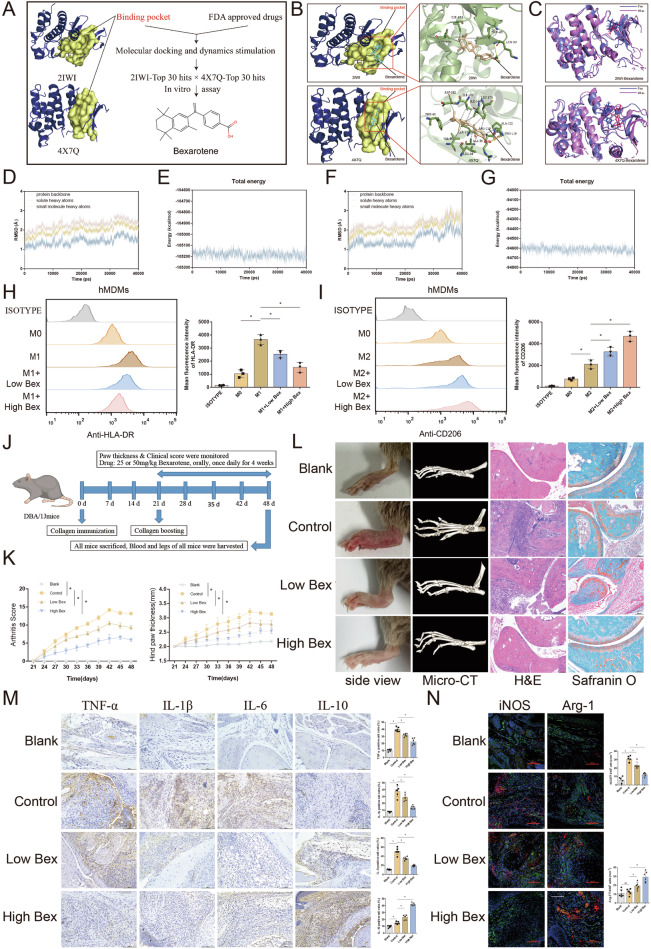
The FDA-approved drug bexarotene inhibits M1 macrophage polarization and alleviates arthritis in CIA model mice. **A** Workflow of the molecular docking and virtual screening analyses. **B** Molecular docking of bexarotene to Pim2 and the bonds between bexarotene and Pim2. **C** Pim2 (2IWI or 4X7Q) interacting with bexarotene at the beginning and end of 40-ns molecular dynamics simulations. **D**, **E** The RMSD and potential energy plot indicating the interaction between Pim2 and bexarotene (PDB 2IWI). **F**, **G** The RMSD and potential energy plot indicating the interaction between Pim2 and bexarotene (PDB ID: 4X7Q). **H**, **I** MFIs of HLA-DR and CD206 in CD68+ hMDMs treated with or without bexarotene (200 or 400 nM) after M1/M2 induction, as detected by flow cytometry (*n* = 3). **J** Schematic of CIA model mice treated orally with bexarotene (25 or 50 mg/kg). **K** CIA scores and hind paw thickness of the mice in each group (*n* = 6). **L** Macroscopic images, micro-CT, H&E, and safranin O staining of the ankles from the mice in each group (*n* = 6). **M** The expression of TNF-α, IL-1β, IL-6 and IL-10 in mice from each group after CIA induction, as detected by IHC. **N** The expression of iNOS and Arg-1 in the synovial macrophages of the mice in each group, as detected by immunofluorescence staining (F4/80, green; iNOS, red; Arg-1, red)

Drugs targeting Pim2 have not yet been approved for clinical use, limiting the application of Pim2 as a therapeutic target for the treatment of human diseases. To identify potential drugs that target Pim2, we performed molecular docking and dynamic simulations via the FDA Drugs Database. Drug-binding scores for the pockets of Pim2 were analyzed via the PROTEIN PLUS website (Supplementary Tables S1 and 2). Various structures of human Pim2, including 2IWI and 4X7Q, have been used for molecular docking. A total of 2,115 FDA-approved drugs were selected for docking analysis. The top 30 drugs with the lowest binding energies in each molecular docking scenario were identified, and 10 drugs—azelastine, bexarotene, dasabuvir, differin, eltrombopag, ergotamine, irinotecan, lumacaftor, nilotinib, and rolapitant—were selected for further analysis (Fig. 7A). The binding energies and rankings of these ten drugs are listed in Supplementary Table S3. To verify the potential of these FDA-approved drugs to target Pim2, we measured the enzymatic activity of Pim2 following incubation with each drug. The results showed that bexarotene inhibited Pim2 activity in a concentration-dependent manner, and its inhibitory effect was slightly weaker than that of HJ-PI01. Nilotinib and irinotecan exhibited slight inhibitory effects, whereas the other seven drugs had no inhibitory effects (Supplementary Fig. S8A).

Next, the interaction between bexarotene and Pim2 was further validated. Visual analysis revealed that bexarotene docks to human Pim2 through hydrogen bonds and hydrophobic bonds, suggesting that bexarotene could influence the ATP binding pocket to inhibit Pim-2 (Fig. 7B). Bexarotene was selected for further molecular dynamic simulations. The root mean square deviation (RMSD) and total energy analyses revealed that the Pim2(2IWI)-bexarotene complex and Pim2(4X7Q)-bexarotene complex were stabilized during the 40 ns simulations (Fig. 7D, E). Furthermore, the structures of the Pim2-bexarotene complexes at the end of the dynamic simulations did not show dramatic changes compared with the original Pim2-drug complex structures (Fig. 7C). These results suggest that bexarotene may specifically target Pim2, indicating its potential for treating inflammatory arthritis.

To explore the effects of bexarotene on the metabolic reprogramming of macrophages in vitro, we initially assessed the glycolytic and oxidative phosphorylation activities in hMDMs treated with bexarotene upon M1 induction. Compared with untreated hMDMs, LPS/IFN-γ-treated hMDMs presented elevated lactate levels and reduced ATP levels, and these effects were reversed by bexarotene (Supplementary Fig. S8B, C). Additionally, compared with untreated hMDMs, LPS/IFN-γ-treated hMDMs presented a greater ECAR and a lower OCR; however, bexarotene reversed these effects (Supplementary Fig. S8D, E). These results indicate that bexarotene promotes the metabolic shift from glycolysis to oxidative phosphorylation in macrophages. Furthermore, we measured the impact of bexarotene on M1/M2 macrophage polarization. Bexarotene significantly reduced the MFI of HLA-DR in LPS/IFN-γ-treated hMDMs but increased the MFI of CD206 in IL-4-treated hMDMs compared with that in untreated hMDMs (Fig. 7H, I). RT‒qPCR analysis revealed that treatment with bexarotene significantly decreased the mRNA levels of IL-1β, IL-6, and TNF-α in the LPS/IFN-γ-treated hMDMs but increased the mRNA levels of IL-10, TGF-β and CD206 in the IL-4-treated hMDMs (Supplementary Fig. S8F, G). Consistently, Western blot analysis revealed that bexarotene reduced the expression of CD86 in hMDMs after M1 induction but increased the expression of CD206 in hMDMs after M2 induction (Supplementary Fig. S8H, I). These results indicate that bexarotene reduces M1 polarization but enhances M2 polarization of macrophages in vitro.

To investigate the therapeutic efficacy of bexarotene in inflammatory arthritis, bexarotene was administered to inflammatory arthritis model mice (Fig. 7J). The CIA score and hind paw thickness were decreased in bexarotene-treated mice (Fig. 7K). Consistently, bexarotene treatment alleviated swelling and redness, decreased inflammatory cell infiltration, and ameliorated cartilage erosion and bone destruction in CIA model mice, as evidenced by H&E staining, safranin O staining, and micro-CT, respectively (Fig. 7L). Additionally, we examined the levels of inflammatory cytokines in the synovial tissues of the mice via IHC staining. Compared with those in the CIA group, the number of TNF-α-, IL-6-, and IL-1β-positive cells was significantly lower in the bexarotene group, whereas the number of IL-10-positive cells was significantly greater (Fig. 7M). Subsequent immunofluorescence staining revealed that the number of iNOS+F4/80 + M1 macrophages was significantly reduced in the bexarotene group, whereas the number of Arg-1 + F4/80 + M2 macrophages was significantly increased (Fig. 7N). These observations indicate that bexarotene reduces M1 polarization but enhances M2 polarization of macrophages in vivo.

Given the well-established role of the retinoid X receptor (RXR) in inflammation and immune regulation by bexarotene [25], we conducted additional genetic and pharmacological experiments to assess whether the observed anti-inflammatory effects are driven predominantly by PIM2 inhibition or RXR activation. In vitro, the RXR-selective agonist LG268 significantly inhibited M1 polarization in BMDMs derived from Pim2^fl/fl^ mice, although its effect was weaker than that of bexarotene. When BMDMs derived from Pim2^fl/fl^-Lyz2^Cre^ mice displayed reduced M1 polarization and enhanced M2 polarization, neither bexarotene nor LG268 appeared to further influence the M1/M2 polarization balance (Supplementary Fig. S9A–F). In vivo, LG268 significantly improved arthritis symptoms in Pim2^fl/fl^ mice, but its therapeutic effect was weaker than that of bexarotene. In Pim2^fl/fl^-Lyz2^Cre^ mice, which presented significantly milder arthritis symptoms, neither bexarotene nor LG268 seemed to provide additional improvement (Supplementary Fig. S9F, G). These results suggest that RXR activation is at least partially involved in the in vitro and in vivo anti-inflammatory effects of BEX.

Taken together, these findings suggest that bexarotene modulates macrophage metabolic reprogramming and M1/M2 polarization through a dual mechanism of synergistically inhibiting Pim2 activity and activating RXR, thereby mitigating the progression of inflammatory arthritis.

### NM@NP-Bex inhibits macrophage M1 polarization and alleviates arthritis in CIA model mice in a targeted and safe manner

Bexarotene is used primarily for the clinical treatment of refractory early-stage cutaneous T-cell lymphoma (CTCL). However, its use is associated with several side effects, including leukopenia, anemia, hyperlipidemia, hypothyroidism, and hepatotoxicity [26]. These adverse effects limit its broader application in treating other diseases. Therefore, we developed a targeted delivery system that can enhance the therapeutic efficacy of bexarotene while minimizing its systemic toxicity and side effects. As shown in Supplementary Fig. S10A, bexarotene was loaded into PLGA-based nanoparticles (NP-Bex). The NPs were coated with neutrophil-derived membranes (NM) via ultrasonication to generate NM@NP-Bex. Compared with that of NP-Bex, the hydrodynamic diameter of NM@NP-Bex increased by approximately 11 nm, indicating successful encapsulation of the neutrophil membrane on the surface of NP-Bex (Supplementary Fig. S10B, C). Additionally, the negative zeta potential of NM@NP-Bex was lower than that of the core (Supplementary Fig. S10D). The drug loading efficiency of NM@NP-Bex was 8.48%, and the encapsulation efficiency was 54.5% (Supplementary Fig. S10E). The drug release curve indicated that NM@NP-Bex also exhibited sustained release of bexarotene (Supplementary Fig. S10F). Dynamic light scattering (DLS) revealed that the size of NM@NP-Bex remained stable in DMEM supplemented with 10% fetal bovine serum (Supplementary Fig. S10G).

The targeted drug loading ability of NMs was achieved through the coordination of various adhesion molecules and chemotactic signaling molecules expressed on the membranes, such as L-selectin, LFA-1, β1-integrin, and CXCR4. To confirm whether these functional adhesive proteins were highly preserved, we performed Western blot analysis. The results revealed four representative adhesion proteins from the NMs in NM@NP-Bex, and their expression significantly increased after LPS stimulation (Supplementary Fig. S10H), which is a prerequisite for the neutrophil mimicry properties of NM@NP-Bex. Overall, these comprehensive features validate NM@NP-Bex as a stable and efficient carrier of bexarotene.

To evaluate the ability of NM@NP-Bex to target inflammatory joints, the biological distribution of Dil-labeled NM@NP-Bex in CIA model mice was measured via an in vivo imaging system (IVIS). Fluorescence was detected in CIA model mice 1 h later and then peaked and remained stable at 6–24 h (Supplementary Fig. S10I). In addition, we detected the fluorescence intensity of various organs in the control group and CIA group after 48 h, and the results revealed significant fluorescence signals in the liver and kidneys of the control and CIA model mice, but there was no significant difference between the two groups. Interestingly, strong fluorescence signals were detected in the ankles of CIA model mice but not in the ankles of control mice (Supplementary Fig. S10J). These results indicate that NM@NP-Bex can target sites of inflammation in the ankles of CIA model mice.

**Fig. 8:**
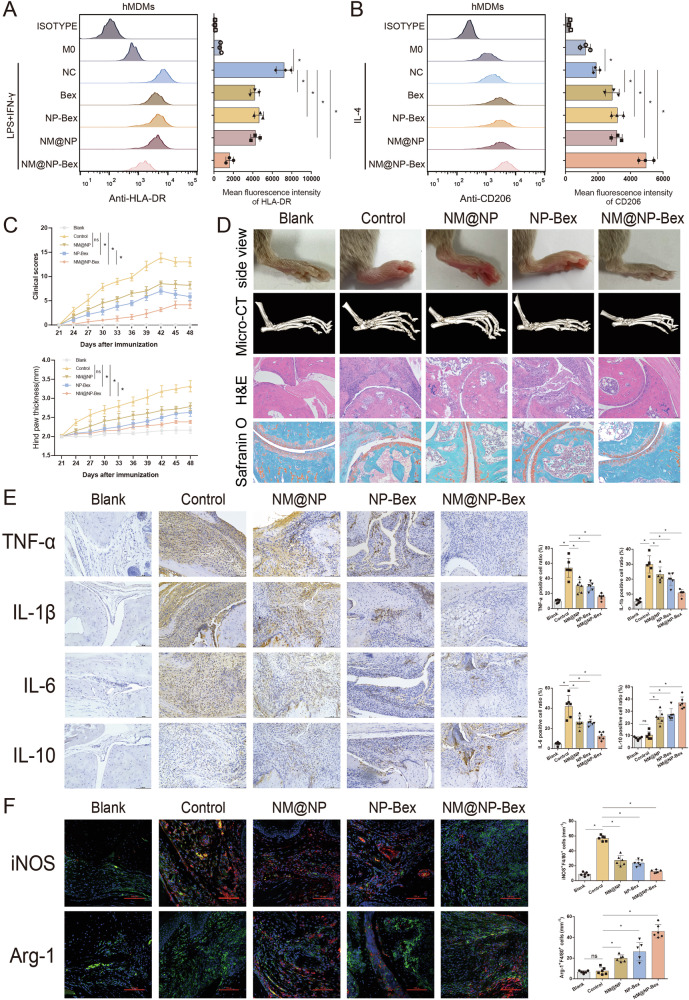
NM@NP-Bex inhibits macrophage M1 polarization and alleviates arthritis in CIA model mice in a targeted and safe manner. **A**, **B** The MFIs of HLA-DR and CD206 were detected in CD68+ hMDMs subjected to different treatments after M1/M2 induction, as determined by flow cytometry (*n* = 3). **C** Arthritis severity was determined via the CIA score (*n* = 6). **D** Macroscopic images, micro-CT, H&E, and safranin O staining of ankles from mice subjected to different treatments (*n* = 6). **E** The expression of TNF-α, IL-1β, IL-6 and IL-10 in mice subjected to different treatments after CIA induction was detected by IHC (*n* = 6). **F** The expression of iNOS and Arg-1 in synovial macrophages from mice subjected to different treatments was detected by immunofluorescence staining (F4/80, green; iNOS, red; Arg-1, red) (*n* = 6)

We next assessed the M1/M2 polarization of macrophages treated with NM@NP-Bex via flow cytometry. Compared with the M1 group, the NP-Bex and NM@NP groups presented a lower MFI of HLA-DR in hMDMs after M1 induction and an increased MFI of CD206 in hMDMs after M2 induction. Notably, the effect of NM@NP-Bex on the M1/M2 polarization of hMDMs was greater than that of NP-Bex or NM@NPs (Fig. 8A, B).

To investigate the therapeutic effect of NM@NP-Bex on inflammatory arthritis in a CIA model, we further tested five treatments in independent groups (healthy mice, PBS-treated CIA mice, blank NM@NP-treated CIA mice, NP-Bex-treated CIA mice, and NM@NP-Bex-treated CIA mice). The CIA score in the NM@NP-Bex group was significantly lower than that in the NM@NP and NP-Bex groups, and the degree of hind paw thickness was the smallest in the NM@NP-Bex-treated group (Fig. 8C). Consistently, NM@NP-Bex treatment most effectively alleviated swelling and redness, decreased inflammatory cell infiltration, and reduced cartilage erosion and bone destruction in CIA model mice, as evidenced by H&E staining, Safranin O staining, and micro-CT (Fig. 8D). These results indicate that NM@NP-Bex treatment is more effective at alleviating inflammatory arthritis than either NM@NP or NP-Bex treatment alone. Additionally, compared with NM@NP or NP-Bex treatment, NM@NP-Bex treatment more effectively inhibited the expression of TNF-α, IL-6, and IL-1β while promoting the expression of IL-10 (Fig. 8E). Finally, the immunofluorescence results revealed that NM@NP-Bex treatment was more effective at reducing the infiltration of M1 macrophages and increasing the infiltration of M2 macrophages in the joint synovium than NM@NP or NP-Bex treatment (Fig. 8F).

To evaluate the biosafety of NM@NP-Bex in vivo, we assessed hepatic and renal toxicity. An increase in the serum triglyceride and total cholesterol levels was observed in the NP-bexarotene-treated CIA model mice but was not detected in the CIA model mice treated with NM@NP-Bex (Fig. S11E, F). Additionally, there were no significant differences in liver or renal function between mice treated with NP-Bex or NM@NP-Bex (Fig. S11A–D, G, H), suggesting that the loading of NM@NPs with bexarotene might be a promising and safe strategy for the treatment of inflammatory arthritis.

In summary, these results indicate that NM@NP-Bex effectively inhibits macrophage M1 polarization and alleviates arthritis in CIA model mice in a targeted and safe manner.

## Discussion

Macrophages constitute the first line of defense in the immune system [27]. Classical M1-type macrophages can directly secrete several proinflammatory factors, such as TNF-α, IL-1β, and IL-6, to establish a proinflammatory microenvironment. Additionally, they can indirectly promote the differentiation of adaptive immune cells, including Th1 and Th17 cells, thereby inducing or maintaining inflammation. In contrast, M2 macrophages, which represent an alternative phenotype, exert anti-inflammatory effects [28–31]. Excessive and persistent M1 polarization, coupled with diminished M2 polarization, is a universal pathological change involved in the aggravation and progression of inflammatory arthritis [4–6]. Consequently, strategies aimed at correcting this M1/M2 macrophage imbalance have become a focal point of research in the treatment of inflammatory arthritis in recent decades.

The metabolic switch from oxidative phosphorylation to glycolysis, even in an oxygen-rich environment, is essential for M1 macrophage polarization and the release of proinflammatory cytokines. M1 macrophages adopt aerobic glycolysis to meet their high demand for quick energy and a sufficient supply of biosynthetic raw materials, fulfilling their needs for proliferation and biosynthesis [7–9]. Additionally, hypoxia, nutrient deficiency, and inflammatory stimuli in the local microenvironment further reprogram energy metabolism from oxidative phosphorylation to glycolysis in macrophages [32, 33]. Therefore, targeting glycolysis inhibition has considerable potential for reversing the M1/M2 macrophage imbalance in the treatment of inflammatory arthritis. Indeed, several studies have explored the possibility of inhibiting glycolysis to treat inflammatory arthritis. Glycolytic inhibitors targeting HK2, PFKFBs, PKM2, and LDHA have been investigated, with some studies even advancing to phase II clinical trials [34–37]. However, to our knowledge, no drugs that target glycolytic inhibition have been approved for clinical use in treating inflammatory arthritis. Thus, the identification of new targets for glycolytic inhibition and their translation into clinical practice is of paramount importance.

Previous studies on Pim2 have focused primarily on its roles in promoting tumor cell survival and preventing apoptosis [14]. In these oncology studies, Pim2 was shown to be a vital kinase for the activation of several glycolytic enzymes [17–20]. However, to our knowledge, a systematic phosphoproteomic analysis exploring the specific role of Pim2 in glycolysis metabolism has not been conducted. In this study, our phosphoproteomic analysis demonstrated that the phosphorylation of the metabolic enzymes PGK1, PDHA1, and PFKFB2 is regulated mainly by Pim2 during M1 polarization. Canonical glycolysis involves a ten-step enzyme-catalyzed reaction, with most glycolytic enzymes having isozymes that perform similar functions [38, 39]. Thus, inhibiting a single enzyme may not be sufficient to block glycolysis in vivo, as other isozymes can compensate for the loss of function. In our study, we demonstrated that Pim2 inhibition or knockdown could simultaneously inhibit the phosphorylation and activation of three different metabolic enzymes: PGK1, PDHA1, and PFKFB2. These enzymes are involved in different steps of glycolysis and the TCA cycle. Therefore, we hypothesize that targeting Pim2 may have greater potential for inhibiting glycolysis in vivo than existing single-enzyme inhibition strategies do. However, there are some discrepancies between our findings and those of previous studies. While PFKFBs were identified as downstream substrates of Pim2, the S203 site of PGK1 and the S300 site of PDHA1 were first discovered in our phosphoproteomic study. Moreover, we could not detect changes in the phosphorylation levels of the glycolytic enzymes HK2 and PKM2 after Pim2 inhibition in macrophages, despite these enzymes being reported as downstream substrates of Pim2 [18, 19]. These findings suggest that the downstream targets of Pim2 involved in regulating glycolysis may vary among different cell types and pathophysiological processes.

Our study further demonstrated that Pim2 is aberrantly overexpressed in M1 macrophages in inflammatory arthritis lesions. Pim2 knockdown in macrophages reprogrammed M1 macrophages to the M2 phenotype, correcting the M1/M2 macrophage imbalance both in vitro and in vivo. Moreover, this reversal of the M1/M2 macrophage imbalance through specific knockout of Pim2 in macrophages significantly suppressed the pathological progression of inflammatory arthritis in a CIA mouse model. In summary, our study successfully demonstrated that Pim2 could be a valuable target for reversing the M1/M2 macrophage imbalance in the treatment of inflammatory arthritis through potent metabolic glycolysis inhibition.

The administration of the selective Pim2 inhibitor HJ-PI01 in vivo significantly reversed the M1/M2 macrophage imbalance and alleviated the severity and progression of CIA, further confirming the therapeutic value of targeting Pim2 in treating inflammatory arthritis. However, to our knowledge, neither pan-Pim inhibitors nor selective Pim2 inhibitors have been approved for clinical application. On the basis of molecular docking and molecular dynamics simulations, bexarotene, a clinically approved drug in the FDA Drugs Database, was shown to bind to Pim2 and inhibit its activity. Additionally, a previous study by Li et al. indicated the potential of bexarotene for treating inflammatory arthritis [25]. Our study further confirmed that bexarotene could at least partially reduce inflammatory cell infiltration and bone and cartilage destruction in CIA model mice via Pim2 inhibition and subsequent reversal of the M1/M2 macrophage imbalance.

Bexarotene is approved by the FDA for the treatment of cutaneous T-cell lymphoma [26]. However, its clinical application is frequently accompanied by multiple side effects. Hyperlipidemia is the most common side effect, occurring in 45–79% of patients. Central hypothyroidism is the second most common adverse effect, occurring in 29–74% of patients. Other adverse effects include leukopenia, headaches, asthenia, and neutropenia [40–42]. Given that inflammatory arthritis is a long-term chronic condition, continuous drug administration is often necessary. To mitigate the adverse side effects of long-term bexarotene use for treating inflammatory arthritis, developing nanocarriers that directly deliver the drug to inflamed regions and reduce off-target effects is crucial. Neutrophil membrane-coated nanoparticles (NM@NPs) are emerging as ideal targeted drug delivery systems for treating inflammatory diseases. Compared with conventional nanocarriers, NM@NPs offer considerable advantages in terms of biocompatibility, stability, specificity, and efficacy. Additionally, the ex vivo production of neutrophils can be used to supply a large quantity of NMs for clinical use [43, 44].

NM@NPs utilize chemokine receptors such as CXCR4 expressed on the NM to migrate to inflamed lesions. They cooperate with β-integrins to adhere to and penetrate inflamed endothelial cells, where they reach sites of inflammation. The rich and varied expression of inflammatory cytokine receptors on the membrane allows NM@NPs, even without drug packaging, to effectively neutralize inflammatory cytokines in arthritis lesions, thereby suppressing inflammation [45–48]. Moreover, active M1 macrophages in arthritis lesions engulf NM@NPs, and the phosphatidylserine expressed on the NM can then polarize M1 macrophages to the M2 phenotype. Moreover, the cytokines and chemokines captured by NM@NPs can be degraded by lysosomes in macrophages [49].

On the basis of these findings, we hypothesized that the use of NM@NPs loaded with bexarotene might be a promising strategy for the treatment of inflammatory arthritis. Our results demonstrated that compared with NP-Bex or NM@NPs alone, NM@NPs loaded with bexarotene had a superior therapeutic effect on reversing the M1/M2 macrophage imbalance and alleviating the severity and progression of CIA in vivo. Moreover, NM@NPs significantly reduced the adverse effects of NP-Bex, including hypertriglyceridemia and hypercholesterolemia, in the CIA mouse model because of their targeted delivery to inflamed joint lesions. In summary, the combination of molecular docking and molecular dynamics simulations with NM@NPs accelerated the use of Pim2 as a clinically viable therapeutic target for treating inflammatory arthritis.

Taken together, the results of our proof-of-principle study indicate that Pim2 might be a valuable target for treating inflammatory arthritis. NM@NPs loaded with bexarotene could represent a promising strategy for the treatment of inflammatory arthritis by effectively inhibiting glycolysis and targeting the M1/M2 macrophage imbalance through Pim2. However, there are still several limitations in our study. Most importantly, we have not elucidated the mechanism underlying the aberrant expression of Pim2 during M1 polarization in inflammatory arthritis. Previous studies have demonstrated that Pim2 expression is commonly regulated by the JAK/STAT signaling pathway. Given that JAK/STAT plays a crucial role in the pathological progression of inflammatory arthritis and that JAK inhibitors have been successfully translated into clinical use for treating autoimmune diseases, including several types of inflammatory arthritis [50–52], we hypothesize that the overexpression of Pim2 in inflammatory arthritis may be due to aberrant activation of the JAK/STAT signaling pathway. Combined blockade of JAK/STAT and its downstream target Pim2 may provide greater therapeutic benefits for patients with inflammatory arthritis. These unresolved scientific issues are of interest for our future research.

## Materials and methods

### Human samples

Synovial tissues were taken from osteoarthritis (OA) and RA patients who underwent artificial total knee joint replacement at the Orthopedic Department of the Eighth Affiliated Hospital, Sun Yat-Sen University. All patients who participated in this study provided informed consent. The study plan was approved by the Ethics Committee of the Eighth Affiliated Hospital, Sun Yat-Sen University. All enrolled patients with RA met the American College of Rheumatology (ACR)/European League Against Rheumatism 2010 classification criteria. The characteristics of the patients with OA and RA are shown in Supplementary Table S4.

### Mice

DBA/1 mice were purchased from GemPharmatech. Pim2^fl/fl^ and Lyz2-Cre mice were purchased from Shanghai Model Organisms Center, Inc. (Shanghai, China). All the mice were bred and maintained under pathogen-free conditions, and age-matched animals were used. The animal experimental procedures were approved by the Institutional Animal Care and Use Committee of Sun Yat-Sen University.

### Establishment of the CIA model

Pim2^fl/fl^ and Pim2^fl/fl^-Lyz2^Cre^ mice aged 6–8 weeks and weighing 20–30 g were subjected to two collagen injections. The initial dose was administered subcutaneously with a mixture of equal parts of chicken type II collagen (Chondrex, #20012) and Freund’s complete adjuvant (Chondrex, #7023). This blend was prepared in a 15 mL centrifuge tube and vigorously mixed at 10,000 rpm for 2 min on ice via a high-speed homogenizer (VRera, #FSH-2A), followed by a 1-min pause. This process was repeated three times to ensure thorough emulsification at low temperatures. A 100 μL sample of the mixture was then injected 2 cm from the base of the tail. The second immunization on day 21 involved a similar procedure in which chicken type II collagen and Freund’s incomplete adjuvant were used (Chondrex, #7002). One hundred microliters of the emulsion was injected slowly into the tail carefully, avoiding areas with enlarged blood vessels from the first injection.

DBA/1 male mice aged 6–8 weeks and weighing 20–30 g underwent two CIA inductions. The initial dose was administered subcutaneously with a mixture of equal parts of chicken type II collagen (Chondrex, #20012) and Freund’s complete adjuvant (Chondrex, #7001). This blend was prepared in a 15 mL centrifuge tube and vigorously mixed at 10,000 rpm for 2 min on ice via a high-speed homogenizer (VRera, #FSH-2A), followed by a 1-min pause. This process was repeated three times to ensure thorough emulsification at low temperatures. A 100 μL sample of the mixture was then injected 2 cm from the base of the tail. The second immunization on day 21 involved a similar procedure in which chicken type II collagen and Freund’s incomplete adjuvant were used (Chondrex, #7002). One hundred microliters of the emulsion was injected slowly into the tail carefully, avoiding areas with enlarged blood vessels from the first injection.

Joint inflammation was assessed every three days and scored as follows: 0 for no change, 1 for mild redness or swelling in one joint, 2 for multiple affected joints, 3 for whole paw inflammation, and 4 for joint ankylosis or deformation. Total scores were calculated for the ankles of each mouse.

### Drug administration in vivo

For the treatment of CIA, DBA/1 mice were treated orally with either 1 mg/mL HJ-PI01 (MCE, #HY-129163), formulated with 0.5% methylcellulose in water, or 1 mg/mL bexarotene (Selleck, #S2098), which contains 0.5% methylcellulose in water. Next, 10 or 20 mg/kg HJ-PI01 and 25 or 50 mg/kg bexarotene were administered orally starting from day 21 until day 42.

For targeted treatment of CIA, DBA/1 mice were given different nanoformulations (NM@NPs, NP-Bex, NM@NP-Bex) at a dose of 1 mg/kg Bex via intravenous injection every 2 days from day 21 until day 48.

### Culture and treatment of BMDMs

BMDMs were isolated from the mice and purified via red blood cell (RBC) lysis buffer (Solarbio, #R1010) to eliminate erythrocytes. The purified cells were then cultured in DMEM supplemented with 10% fetal bovine serum (FBS) and 10 ng/ml recombinant mouse M-CSF (Sino Biological, #51112) for 7 days to facilitate their differentiation into macrophages. Following this period, to induce M1 polarization, M0 macrophages were exposed to 100 ng/ml LPS (Sigma, L2880) and 50 ng/ml IFN-γ (PeproTech, 315-05). For M2 polarization, M0 macrophages were treated with 10 ng/ml each of IL-4 (PeproTech, 214-14) and IL-13 (Beyotime, P5948).

### Isolation and culture of human monocyte-derived macrophages (hMDMs)

Human peripheral blood was mixed with an equal volume of Ficoll separation solution (TBD, #LDS1075) and centrifuged via a density gradient at 400 × *g* to separate the components. Peripheral blood mononuclear cells (PBMCs) were harvested from the interface layer, washed twice with PBS, and then quantified. The PBMCs were resuspended in serum-free RPMI 1640 medium (Gibco, C11875500BT), and 5 × 10^6^ cells were plated per well in a 12-well plate, followed by incubation at 37 °C for 4 h. After incubation, the supernatant was removed, and the cells were gently washed with PBS. The cells were then cultured in RPMI 1640 medium supplemented with 10% FBS and 25 ng/ml M-CSF (PeproTech, 300--25--10) for 5 days, and the medium was changed every three days. For M1 macrophage differentiation, 50 ng/ml LPS (Sigma, L2880) and 25 ng/ml IFN-γ (PeproTech, 300-02-20) were added to M0 macrophages on day 6, and the cells were harvested after an additional 48 h of culture for further analysis. To generate M2 macrophages, 20 ng/ml IL-4 (Peprotech, 200--04--5) and 20 ng/ml IL-13 (Peprotech, 200--13--2) were added to M0 macrophages on day 6, and the cells were similarly collected after 48 h of cultivation for subsequent experiments. For the indicated experiments, HJ-PI01 (100 or 200 nM), bexarotene (200 or 400 nM), NP-Bex (400 nM), NM@NP-Bex (400 nM) and NM@NPs (the equivalent of 400 nM NM@NP-Bex) were added during M1/M2 polarization.

### RNA sequencing and data analysis

Untreated hMDMs (M0 group), LPS/IFN-γ-treated hMDMs (M1 group) and LPS/IFN-γ- and HJ-PI01-treated hMDMs (M1H group) were collected, and total RNA was extracted via a previously described method. The PolyA+ RNA fraction was then processed, after which the cDNA library was constructed. The Beijing Genomics Institute sequenced the samples via the BGISEQ500 platform. The thresholds for differential expression analysis were a fold change of at least 1.5 and an adjusted P value of 0.05 or less. Comprehensive sequencing data analysis was carried out with the BGI Dr. Tom 2.0 webtool, which included heatmap clustering, the generation of Venn diagrams, GO analysis, KEGG analysis, and GSEA.

### OCR and ECAR measurements

The Seahorse XF Cell Mito Stress Test Kit (Seahorse, #103015-100) and Seahorse XF Glycolysis Stress Test Kit were utilized for the determination of the OCR and ECAR, respectively. PBMC-derived macrophages (1 × 10^4^) were seeded in each well of an XF96 plate. Following the specified treatment protocols, the cells were analyzed via a Seahorse XFe96 analyzer (Agilent) to assess the OCR and ECAR. For mitochondrial stress, the cells were sequentially exposed to 1 μm oligomycin, 1 μm carbonyl cyanide p-trifluoromethoxyphenylhydrazone (FCCP), and 150 nm rotenone. The oxygen levels were recorded after the addition of each compound, and the results were normalized to the protein concentration of each well. The basal OCR was determined by the difference in the OCR readings before and after oligomycin introduction, whereas the maximum OCR was calculated as the difference between the readings after FCCP addition and those after rotenone addition. The ECAR was evaluated by sequentially treating the cells with 10 mm glucose, 1 μm oligomycin, and 50 mm 2-deoxy-glucose (2-DG). The impact of each addition on the ECAR was measured, and the data were again normalized to the well protein concentration. The basal ECAR was determined by the difference in the readings before and after glucose addition, whereas the maximum ECAR was defined as the difference between the readings before glucose addition and those after oligomycin treatment.

### Measurement of lactate production

Lactate levels were quantified via a lactic acid assay kit (Abbkine, #ktb1100) according to the manufacturer’s instructions. In summary, 50 μL of each cell culture supernatant was combined with 50 μL of lactate assay buffer and an equal volume of working reagent in a 96-well plate. This mixture was then incubated at 37 °C for 30 min in the dark. Following incubation, the absorbance of each well at 450 nm was recorded via a luminometer (BIOTEK Synergy HTX). Lactate production was then normalized against the protein concentration in each sample.

### ATP measurement assay

Cellular ATP concentrations were determined via an ATP Assay Kit (Beyotime, #S0026). The cells were lysed, and the lysates were centrifuged at 12,000 × *g* for 5 minutes at 4 °C. Subsequently, 20 μL of the resulting supernatant was mixed with 180 μL of ATP detection working buffer. This mixture was then dispensed into a 96-well plate. ATP levels were quantified via a luminometer (BIOTEK Synergy HTX), and the results are expressed in relative light units and were normalized to the protein concentration in each sample.

### Mass spectrometry data analysis

M1 macrophages, both with and without 200 nM HJ-PI01 treatment, were collected and lysed in a solution containing 8 M urea, 1% protease inhibitors, 1% phosphatase inhibitors, 3 μM trichostatin A, and 50 mM nicotinamide via sonication at 4 °C. After sonication, the lysates were centrifuged at 12,000 × *g* for 10 min at 4 °C to separate the supernatant. The protein concentrations in the supernatants were then quantified via a BSA assay kit (CWBIO, #CW0014s). Proteins were precipitated by incubation with 20% trichloroacetic acid (TCA) for 2 hours at 4 °C, followed by washing with acetone and air drying the precipitates. The dried samples were resuspended in 200 mM TEAB, and trypsin was added at a 1:50 protease-to-protein mass ratio overnight enzymatic digestion at 4 °C. The peptides were subsequently reduced with 5 mM dithiothreitol for 30 min at 56 °C and alkylated with 11 mM iodoacetamide for 15 min in the dark at room temperature. The peptides were then purified via a Strata X C18 SPE column (Agela-Phenomenex, #00B-S001-A0) and lyophilized under vacuum. Finally, the peptides were reconstituted, separated via a NanoElute ultrahigh-performance liquid chromatography system, and analyzed via mass spectrometry with an NSI ionization source. The electrospray ionization voltage was configured to 1.75 kV, and the mass range for secondary mass spectrometry analysis was defined from 400 to 1500 m/z. Data acquisition was conducted in parallel cumulative serial fragmentation (PASEF) mode, involving a sequence of one primary MS acquisition followed by 10 secondary MS acquisitions in PASEF mode, with a dynamic exclusion period of 30 s. Protein identification and posttranslational modification (PTM) site quantification were performed via the MaxQuant search engine (version 1.5.2.8). The allowable number of missed cut sites was set to 2. The peptide length cutoff was set as 7 amino acid residues, and the maximum number of peptide modifications was set as 5. The mass error tolerances for the primary parent ion and the main search were set as 20 and 5 ppm, respectively. The mass error tolerance of the secondary fragment ion was set as 0.02 Da. The identifications were filtered to a 1% FDR. Analytical processing of the data utilized tools such as InterProScan, KEGG Mapper, WoLF PSORT, CELLO, and the R package pheatmap.

### RNA interference

To knock down specific genes, both targeting and control siRNAs were obtained from GenePharma. For transfection, 0.2 nmol of siRNA was combined with 4 μL of Lipofectamine RNAiMAX (Invitrogen, #13778150) in 100 μL of Opti-MEM reduced serum medium (Thermo Fisher, #31985070) and incubated separately at 37 °C for 5 min. After this initial incubation, the siRNA and Lipofectamine RNAiMAX mixtures were combined, allowed to incubate for an additional 20 min at 37 °C, and then applied to the target cells. After transfection, the cells were maintained in reduced-serum Opti-MEM for 6 h, after which the medium was changed to DMEM supplemented with 10% FBS. The effectiveness of the gene silencing was assessed three days later, after which the cells were subjected to further experiments. Control experiments utilized nontargeting siRNA. The sequences of all the siRNAs used are detailed in Supplementary Table S5.

### RNA preparation and RT‒qPCR

Total RNA was extracted from cells via TRIzol reagent (Invitrogen, #15596--026) and then reverse transcribed with Evo M-MLV RT Master Mix (AG, #11706) following the manufacturer’s instructions. RT‒qPCR analysis was conducted via the SYBR Green Premix Pro Taq HS qPCR Kit (AG, #11718). Gene expression quantification was performed via the 2 − △△Ct method, with normalization to β-actin expression levels. The primer sequences utilized for amplifying each gene are listed in Supplementary Table S6.

### Western blotting analysis

The cells were lysed via RIPA buffer (Sigma‒Aldrich, # R0278) supplemented with 1% protease (CWBIO, #CW2200S) and phosphatase inhibitors (CWBIO, #CW2383S) and maintained at 4 °C for 30 min. After centrifugation at 11,000 × *g* for 30 min at 4 °C, the supernatant was extracted, and the protein concentration was quantified via a BCA protein assay kit (CWBIO, #CW0014S) according to the supplier’s instructions. Following preparation with Beyotime sample loading buffer (#P0015) and boiling, the supernatant was used for immunoprecipitation. Initially, the supernatant was cleared with protein A/G beads (Invitrogen, #88802) before overnight antibody incubation at 4 °C. After a 3-h bead incubation, the immunoprecipitates were washed, prepared with loading buffer, and boiled. Proteins were then resolved by SDS‒PAGE and transferred to PVDF membranes (Millipore, #IPVH00010). The membranes were blocked with 5% BSA and incubated with primary antibodies overnight at 4 °C, followed by incubation with HRP-conjugated secondary antibodies for 1 h, and detection was facilitated by enhanced chemiluminescence (ECL) (Millipore, #WBKLS0500). The antibodies used are listed in Supplementary Table S7.

### Pim2 activity measurement

Pim2 kinase activity was assessed via a commercial assay kit (Promega, #V4035). The assay was performed by mixing 1 μL of a specified inhibitor with 2 μL of Pim2 enzyme and 2 μL of substrate/ATP mixture, followed by incubation at room temperature for 60 min. Subsequently, 5 μL of ADP-Glo™ Reagent was added, and the mixture was further incubated for 40 min. To complete the reaction, 10 μL of Kinase Detection Reagent was added, and the mixture was incubated for an additional 30 min at room temperature. Pim2 activity was quantified by measuring relative luminescence with a luminometer.

### Molecular docking

Structural analyses of Pim2 (PDB IDs: 2IWI, 4X7Q) were conducted via data retrieved from the RCSB Protein Data Bank (www.rcsb.org). Using PyMOL (version 2.5.3), cocrystallized ligands and heteroatoms were excised, and water molecules were removed. Hydrogens were added to the structures with AutoDockTools (version 1.5.7). FDA-approved molecular structures in mol2 format were sourced from the ZINC database (zinc.docking.org) and converted to PDB via Open Babel (version 3.3.1), where hydrogen atoms were appended and charges were computed. Docking simulations were performed with AutoDockTools, prioritizing molecules on the basis of the lowest computed binding energies. Interactions between Pim1 and these molecules during docking were investigated via the protein‒ligand interaction profiler (plip-tool.biotec.tu-dresden.de), and the resulting Pim2-drug complexes were visualized via PyMOL.

### Molecular dynamics simulation

The structures of Pim2 (PDB IDs: 2IWI, 4X7Q) were prepared by removing water molecules and cocrystallizing ligands via PyMOL. Missing atoms were restored with SPDBV (version 4.1.0). The processed structures were then utilized in molecular dynamics simulations conducted via GROMACS (2022.2) with the AMBER99SB-ILDN force field. The Pim2 structures were converted to topological formats, and ligand structural data were incorporated. A simulation box was constructed ensuring a minimum distance of 1.2 nm from the protein to the box edge. Initially, the system’s energy was minimized in vacuum, followed by solvation and ion addition, with subsequent energy minimization. After position-restrained equilibration, a 10-ns production simulation was carried out. The RMSD values were quantified via the g_rms, g_rmsdist, and g_rmsf tools. Visualization of the Pim2-drug complexes postsimulation was performed via PyMOL.

### Micro-CT scanning

Mouse ankle joints were collected and preserved in 4% paraformaldehyde before being subjected to micro-CT scanning with a Siemens Inveon CT scanner. Three-dimensional reconstructions of the scanned images were executed via RadiAnt DICOM Viewer software (version 5.0.2).

### Cell immunofluorescence staining

The cells on the confocal dishes were fixed with 4% paraformaldehyde, permeabilized with 0.5% Triton X-100, and blocked with 5% BSA. The membranes were then incubated with primary antibodies overnight at 4 °C, followed by incubation with fluorescein-labeled secondary antibodies for 1 h at room temperature in the dark. Confocal images were acquired with a Nikon Eclipse Ni-E imaging system. The primary antibodies used are listed in Supplementary Table S7.

### Histological staining

Harvested samples were fixed in 4% paraformaldehyde for 24 h, decalcified in 50 mM EDTA for two weeks, and subsequently embedded in paraffin. The tissue sections were then deparaffinized and rehydrated through sequential immersion in xylene followed by a graded series of ethanol solutions.

### H&E staining

Rehydrated sections were stained with hematoxylin (Servicebio, #G1004) for 10 min, followed by immersion in 1% alcoholic hydrochloric acid for 5 s and rinsing with PBS. The sections were then subjected to eosin staining (BOSTER, #AR1180-2) for 2 min. The sections were washed, dehydrated, and mounted with neutral resin. Imaging was performed via a standard microscope. Arthritis inflammation was assessed on H&E-stained sections via a graded scoring system: 0, normal joint; 1, mild thickening of the lining layer or mild inflammatory cell infiltration; 2, both mild thickening and inflammatory cell infiltration; 3, moderate thickening and inflammatory cell infiltration, with cells in the synovial space; and 4, severe inflammatory cell infiltration in the synovium.

### Safranin O‒Fast Green staining

Safranin O-Fast Green staining was conducted via a modified kit (Solarbio, #G1371). Rehydrated sections were subjected to Weigert staining for 5 min, followed by acidic differentiation for 15 s. Fast green staining was applied for 5 min, with subsequent weak acidic differentiation for 10 s. After drying, the sections were stained with safranin O for 5 min, dehydrated, and mounted with neutral resin. Microscopy was used for image acquisition. Cartilage damage was evaluated on stained sections with a scoring system: 0 represented a normal joint; 1 indicated partial erosion of the cartilage surface; 2 denoted erosion along with cartilage destruction; and 3 described extensive cartilage erosion and destruction overlaid by connective tissue.

### Tissue immunofluorescence staining

The samples were fixed in 4% paraformaldehyde for 24 h, decalcified with 50 mM EDTA for two weeks, and embedded in paraffin. The sections were deparaffinized, rehydrated, and permeabilized with 0.5% Triton X-100 for 30 min. Antigen retrieval was achieved by heating in 10 mM citrate buffer at 750 W for 20 min in a microwave. After cooling to room temperature, the sections were blocked with 5% normal goat serum and incubated overnight with primary antibodies at 4 °C. The following day, after being washed with PBS, the sections were incubated with fluorescein-labeled secondary antibodies for 1 h at room temperature in the dark. Confocal images were captured using a Nikon Eclipse Ni-E system.

### Tissue immunohistochemical staining

Following the previously described procedures for sample preparation, the sections were deparaffinized, rehydrated, and subjected to antigen retrieval by microwaving in 10 mM citrate buffer at 750 W for 20 min. After cooling to room temperature, the sections were treated with 3% H2O2 for 25 min to quench endogenous peroxidases, followed by blocking with 5% normal goat serum for 30 min. Overnight incubation at 4 °C was then performed with primary antibodies. The next day, the sections were incubated with secondary antibodies and developed via an SP Rabbit & Mouse HRP Kit (CWBIO, #CW2069S). Imaging was conducted via a microscope. The primary antibodies used are listed in Supplementary Table S7.

### Flow cytometry

To assess M1/M2 macrophage polarization, the collected hMDMs/BMDMs were incubated with the following specific antibodies: antihuman HLA-DR-PE (BD, #555812), antimouse MHC-II-FITC (BD, #562009), antihuman CD206-APC (BD, #550889) and F4/80-PE (BD, #565410) for 30 min at room temperature. After three washes, the cells were treated with a fixation/permeabilization solution (eBioscience, #00--5523--00) in the dark for 60 min. Following another three washes, the cells were incubated with an anti-human CD68-FITC antibody (BD, #562117) and anti-mouse CD206-Alexa Fluor 647 (BD, #565250) for 30 min in the dark. The cells were then resuspended in 200 µL of PBS and transferred to tubes for analysis with a BD FACSCelesta flow cytometer.

### Isolation and activation of peripheral neutrophils

Neutrophils were isolated from the whole blood of male ICR mice via the Percoll gradient method (Boxio et al.). Blood was collected in heparin tubes and centrifuged (400 × *g*, 10 min, 4 °C). The resulting cell pellets were resuspended in PBS with EDTA, layered onto a three-tiered Percoll gradient (52%, 69%, 78%) and centrifuged at 1500 × *g* for 30 min at room temperature (GE Healthcare Bio-Sciences AB, Uppsala, Sweden). Neutrophils were collected from the 69/78% interface and the upper 78% layer, followed by erythrocyte lysis at 4 °C. For activation, neutrophils were incubated with lipopolysaccharide (LPS, 100 ng/mL) for 4 h and washed in PBS.

### Isolation of neutrophil plasma membranes

Plasma membranes were isolated from LPS-stimulated neutrophils following the protocol of Pilchler et al. Cells were suspended in ice-cold isolation buffer-1 (IB-1) containing 225 mM mannitol, 75 mM sucrose, 0.5% (w/v) BSA, 0.5 mM EDTA, and 30 mM Tris-HCl supplemented with a protease inhibitor cocktail. The suspension was homogenized via a dounce homogenizer with a pestle for 50–100 strokes. The homogenate was centrifuged at 800 × *g* for 10 min at 4 °C to pellet unbroken cells and nuclei. The resulting supernatant was further centrifuged at 10,000 × *g* for 10 min at 4 °C to sediment the mitochondria, after which the pellet was discarded. The final supernatant was centrifuged at 100,000 × *g* for 1 h at 4  °C. The resulting plasma membrane pellet was washed in 10 mM Tris-HCl and 0.5 mM EDTA with protease inhibitor cocktail, freeze-dried, weighed, and stored at −80 °C for subsequent analysis.

### Preparation of the PLGA-PEG@Bexarotene NPs

Bexarotene-loaded polymer NPs were prepared via a modified oil-in-water (O/W) emulsion solvent evaporation method. First, 500 mg of the PLGA-PEG copolymer and 50 mg of bexarotene were dissolved in a mixture of the organic solvents dichloromethane (2.5 mL, immiscible with water) and acetone (2.5 mL, miscible with water). The resulting solution was slowly added to 50 mL of distilled water via a pipette. The mixture was emulsified into nanodroplets via a probe sonicator (Xinzhi Biotechnology Co., Ltd., China) on ice with an output of 180 W for 45 s. Dichloromethane and acetone were evaporated under reduced pressure via a rotary evaporator at 30 °C for 3 h. The dispersed nanodroplets were then solidified in an aqueous solution. The entire dispersed system was filtered through a membrane filter (pore size: 0.45 μm, SLHP033RB, Millipore) to separate the NPs. Blank nanoparticles were prepared without the addition of bexarotene.

### Preparation of NM@NP-Bex

NMs were dissolved in PBS, repeatedly freeze-thawed twice and sonicated for 5 min in liquid nitrogen and at room temperature to obtain a hydrated cell membrane suspension. The prepared PLGA-PEG@Bexarotene NPs were dispersed in PBS. The weight ratio of the nanoparticles to the membrane protein was 1:1. Using an Avanti microextruder, the suspension was sequentially passed through a polycarbonate porous membrane with pore sizes of 800 nm, 400 nm, and 200 nm (ultimately depending on the particle size of the PLGA-PEG@Bexarotene NPs) and extruded 15 times to prepare the NM@NP-Bex. The prepared NM@NP-Bex were stored at 4 °C for later use.

### Identification of membrane-associated proteins

Membrane-associated proteins on the particles were assessed via Coomassie blue staining and Western blot analysis. NM@NPs were isolated by centrifugation at 14,500 rpm for 50 min at 4 °C to remove the uncoated NMs. Samples including prehomogenized neutrophils, NMs, and NM@NPs were lysed via RIPA buffer (50 mM Tris, pH 7.4; 150 mM NaCl; 1% Triton X-100; 1% sodium deoxycholate; 0.1% SDS) supplemented with protease inhibitor cocktail (cOmplete, Roche, Germany) on ice for 5 min. Lysates were subsequently centrifuged at 13,000 × *g* for 5 min at 4 °C, after which the protein concentration in the supernatant was quantified via an enhanced BCA assay (Beyotime Biotechnology, Haimen, China). Proteins were then resolved by SDS‒PAGE and transferred to PVDF membranes (Millipore, #IPVH00010). The membranes were blocked with 5% BSA and incubated with primary antibodies overnight at 4 °C, followed by incubation with HRP-conjugated secondary antibodies for 1 h, and detection was facilitated by enhanced chemiluminescence (ECL) (Millipore, #WBKLS0500). The antibodies used are listed in Supplementary Table S7.

### Statistical analysis

The data are presented as scatter plots displaying the means ± standard deviations. Statistical analyses were conducted via GraphPad Prism 8.0 (GraphPad Prism Software, CA, USA). Sample normality was assessed via the Shapiro‒Wilk test. Differences between independent samples were evaluated via two-tailed Student’s *t* tests, and paired t tests were used for paired samples. Group comparisons (of three or more groups) were performed via one-way ANOVA with Bonferroni post hoc correction. Additionally, two-way repeated-measures ANOVA was employed to assess differences in CIA scores across groups. The sample sizes (n) are indicated in the figure legends. A *p* value threshold of 0.05 was established for statistical significance.

## Supplementary information

Supplementary materials

unprocessed original images
