# Supplementary material for: Targeting macrophage polarization by inhibiting Pim2 alleviates inflammatory arthritis via metabolic reprogramming
Source: Cell Mol Immunol. 2025 Feb 26;22(4):418–36. doi: 10.1038/s41423-025-01268-9 (PMC11955556; doi:10.1038/s41423-025-01268-9)
Supplement: Supplementary file 1 — Supplementary materials [file 41423_2025_1268_MOESM1_ESM.docx]

**Supplementary materials for**

**Targeting macrophage polarization by inhibiting Pim2 alleviates inflammatory arthritis via metabolic reprogramming**

Xiaojun Xu^1,3*^, Peitao Xu^1,3*^, Guozhen Shen^1,3*^, Xiaoshuai Peng^1,3^, Zhidong Liu^1,3^, Chaoqiang Chen^1,3^, Wenhui Yu^1,3^, Zepeng Su^1,3^, Jiajie Lin^1,3^, Guan Zheng^1,3^, Guiwen Ye^1,3^, Peng Wang^1,3^, Zhongyu Xie^1,3^, Yanfeng Wu^2,3#^, Huiyong Shen^1,3#^, Jinteng Li^1,3#^

Correspondence: Jinteng Li(lijt57@mail.sysu.edu.cn); Huiyong Shen([shenhuiy@mail.sysu.edu.cn](mailto:shenhuiy@mail.sysu.edu.cn)); Yanfeng Wu([wuyf@mail.sysu.edu.cn](mailto:wuyf@mail.sysu.edu.cn))

**This file includes:**

Supplementary Figures 1 to 13

Supplementary Tables 1 to 7

**Supplementary Figures**

**
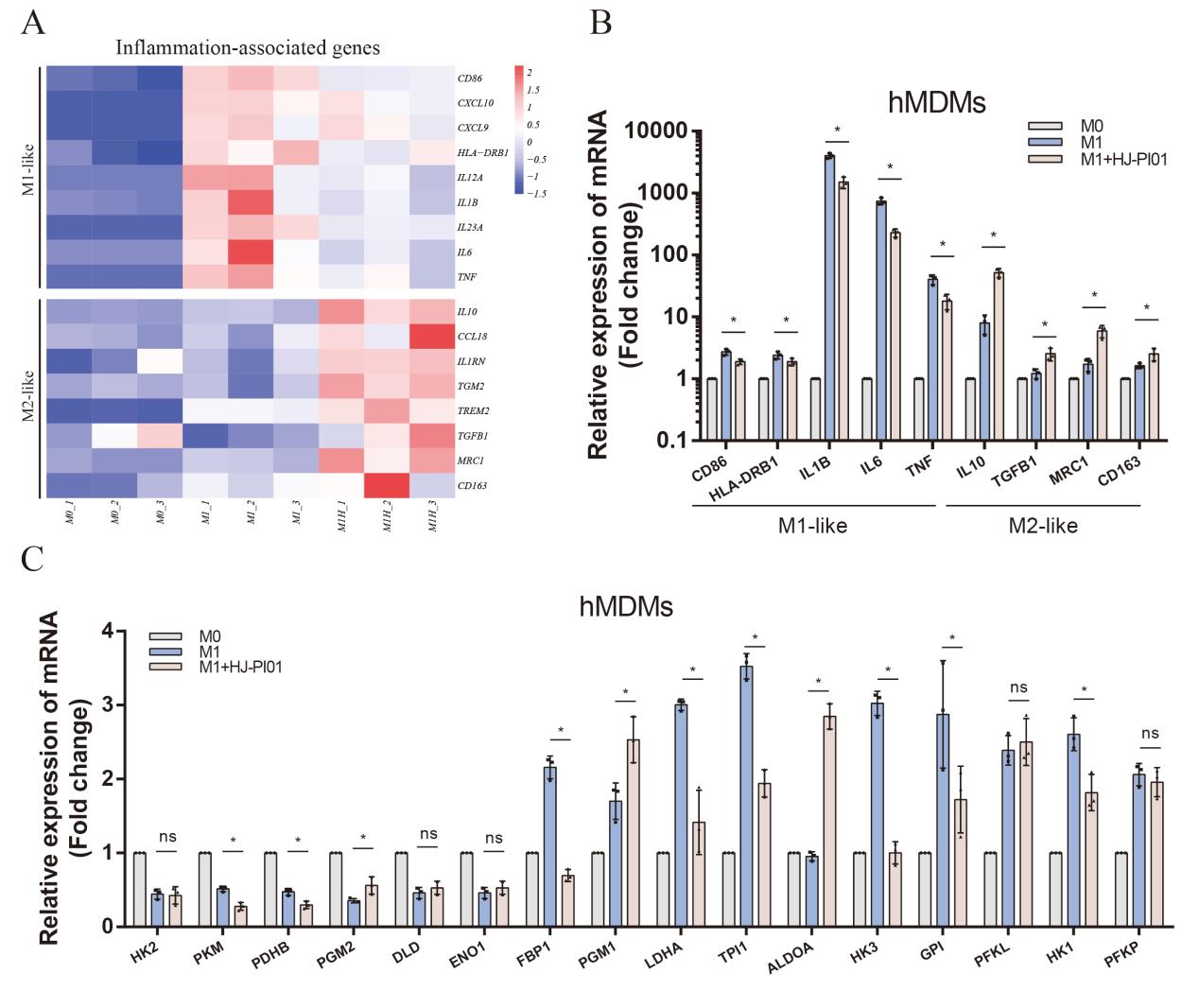
**

**Fig. S1. Validation of inflammation and glycolysis-associated genes regulated by HJ-PI01**

1. Heatmap of inflammation-associated DEGs identified by RNA-seq in the M0, M1, and M1H groups. B-C. The mRNA level of inflammation and glycolysis-associated genes in the M0, M1 and M1H groups, as determined by RT-qPCR (n=3).

**
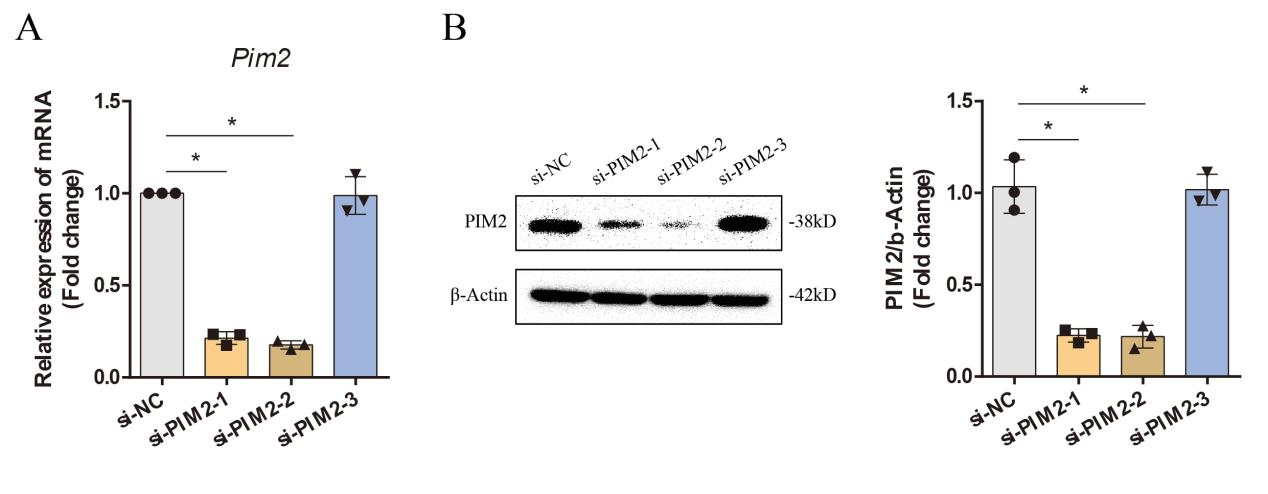
**

**Fig. S2. Knockdown efficiency of siRNAs**

A-B. The knockdown efficiency of Pim2 by siRNAs was detected by RT‒qPCR and Western blotting (n=3).


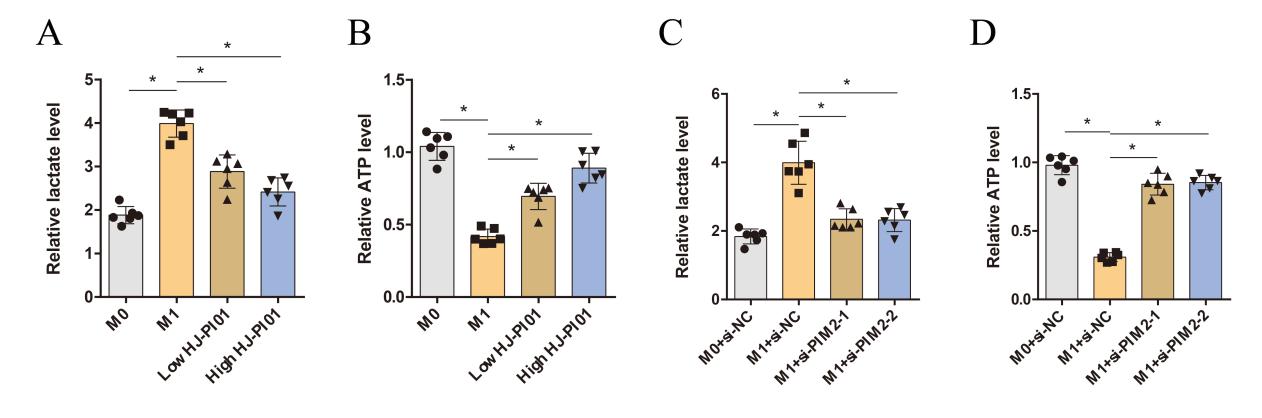


**Fig. S3. Pim2 promotes glycolytic reprogramming in M1 macrophages**

A-B. Relative levels of ATP and lactate in hMDMs treated with or without HJ-PI01(100 or 200 nM) after M1 induction (n=6). C-D. Relative levels of ATP and lactate in hMDMs in the presence or absence of Pim2 knockdown after M1 induction (n=6).


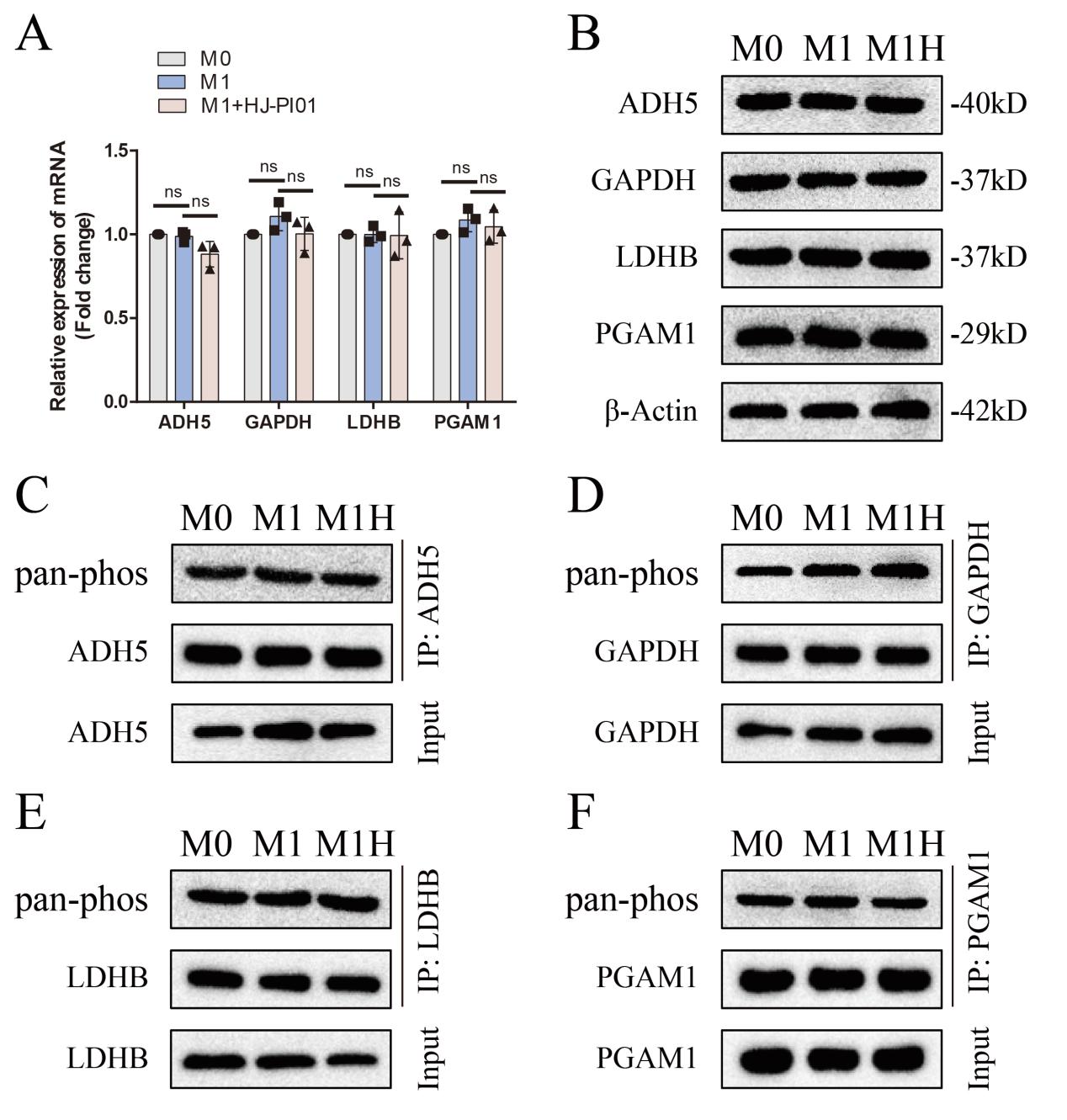


**Fig. S4. Expression level and phosphorylation level of glycolytic enzymes**

A-B. The mRNA and protein levels of ADH5, GAPDH, LDHB and PGAM1 in hMDMs with or without 200 nM HJ-PI01 treatment after M1 induction were determined by RT‒qPCR and Western blotting (n=3). C-E. The phosphorylation levels of ADH5, GAPDH, LDHB and PGAM1 in hMDMs with or without 200 nM HJ-PI01 treatment after M1 induction were determined by immunoprecipitation followed by Western blotting (n=3).


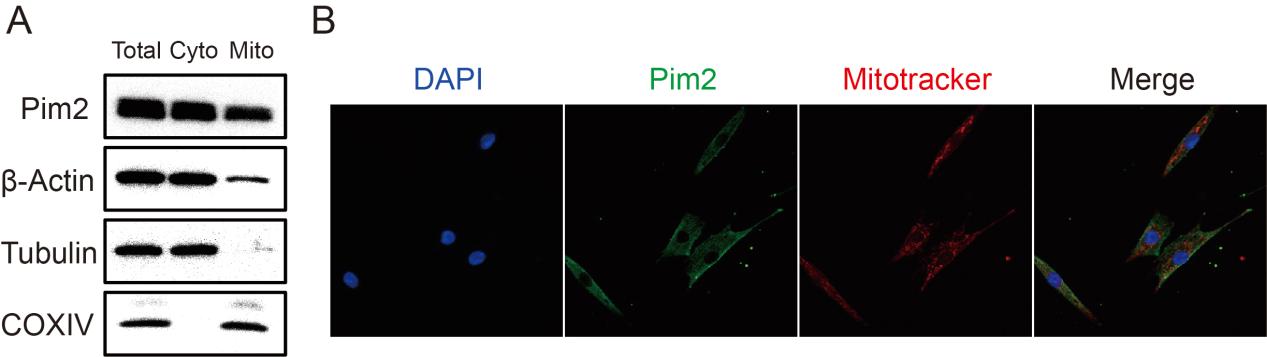


**Fig. S5. The colocalization of Pim2 and mitochondria.**

1. The expressions of Pim2 in total, cytoplasmic, and mitochondrial fractions of 293T cells were assessed. β-actin, Tubulin, and COXIV were served as internal references for the total, cytoplasmic, and mitochondrial fractions, respectively (n=3). B. Immunofluorescence was used to detect the colocalization of Pim2 and mitochondria in 293T cells. Pim2 was labeled with green fluorescence, while mitochondria were stained with red fluorescence (n=3).


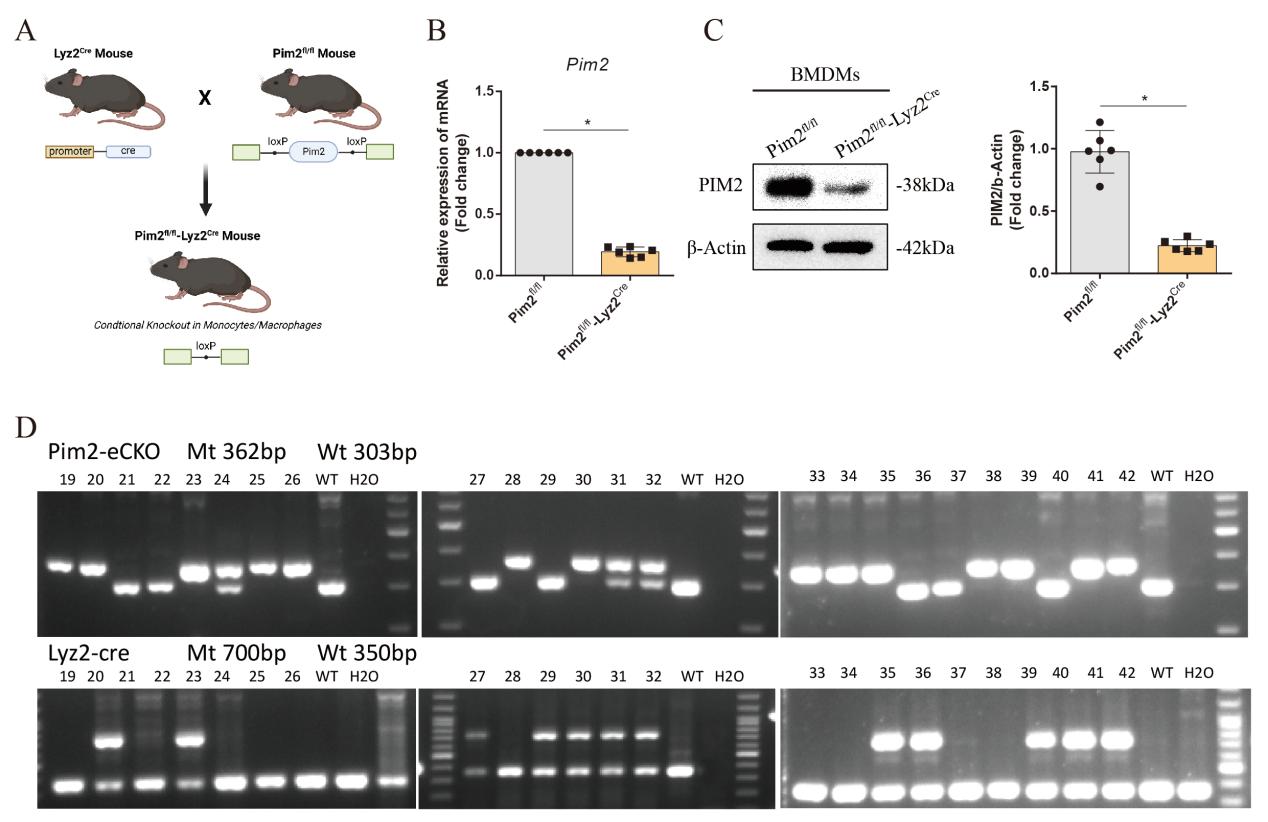


**Fig. S6. Construction and validation of Pim2^fl/fl^-Lyz2^Cre^ mice**

A. Construction strategy for Pim2^fl/fl^-Lyz2^Cre^ mice. B. The mRNA and protein levels of Pim2 in BMDMs from Pim2^fl/fl^ and Pim2^fl/fl^-Lyz2^Cre^ mice, as detected by RT‒qPCR and Western blotting (n=6).


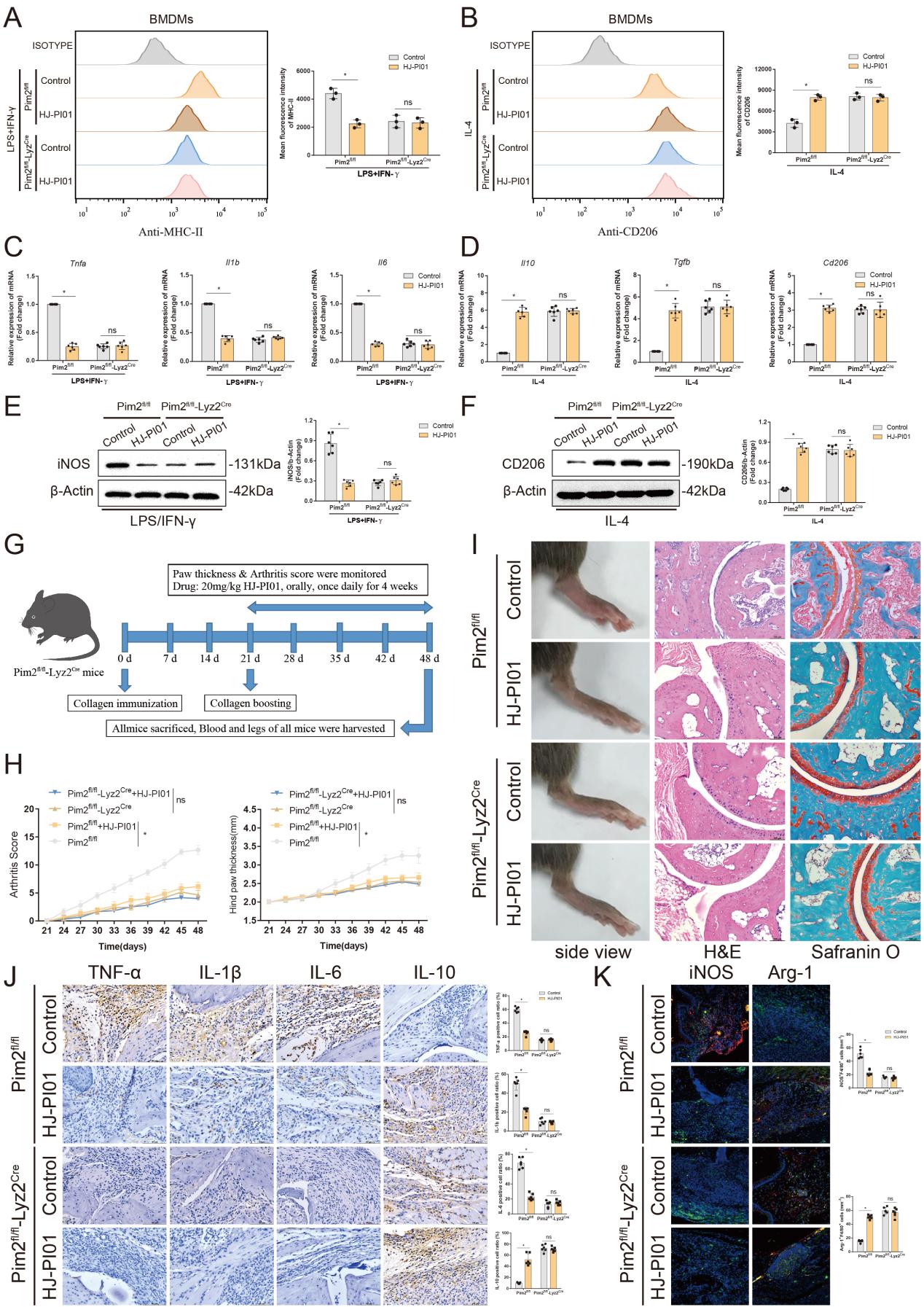


**Fig. S7. HJ-PI01 exerts its regulatory effect on macrophage polarization by targeting PIM2 inhibition, thereby improving inflammatory arthritis.**

A-B. The MFI of MHC-II and CD206 in F4/80+ BMDMs treated with or without HJ-PI01(200 nM) after M1/M2 induction , as detected by flow cytometry (n=6). C-F. The mRNA and protein expression levels of inflammatory factors in BMDMs treated with or without HJ-PI01(200 nM) after M1/M2 induction, as determined by RT‒qPCR, Western blotting (n=6). G. Schematic of CIA model mice treated with HJ-PI01(20 mg/kg). H. CIA scores and hind paw thickness of mice in each group (n=6). I. Macroscopic images, H&E, and Safranin O staining of the ankles from mice in each group (n=6). J. The expression of TNF-α, IL-1β, IL-6 and IL-10 in mice in each group, as detected by IHC (n=6). K. The expression of iNOS and Arg-1 in the synovial macrophages of mice in each group, as detected by immunofluorescence staining (F4/80, green; iNOS, red; Arg-1, red) (n=6).


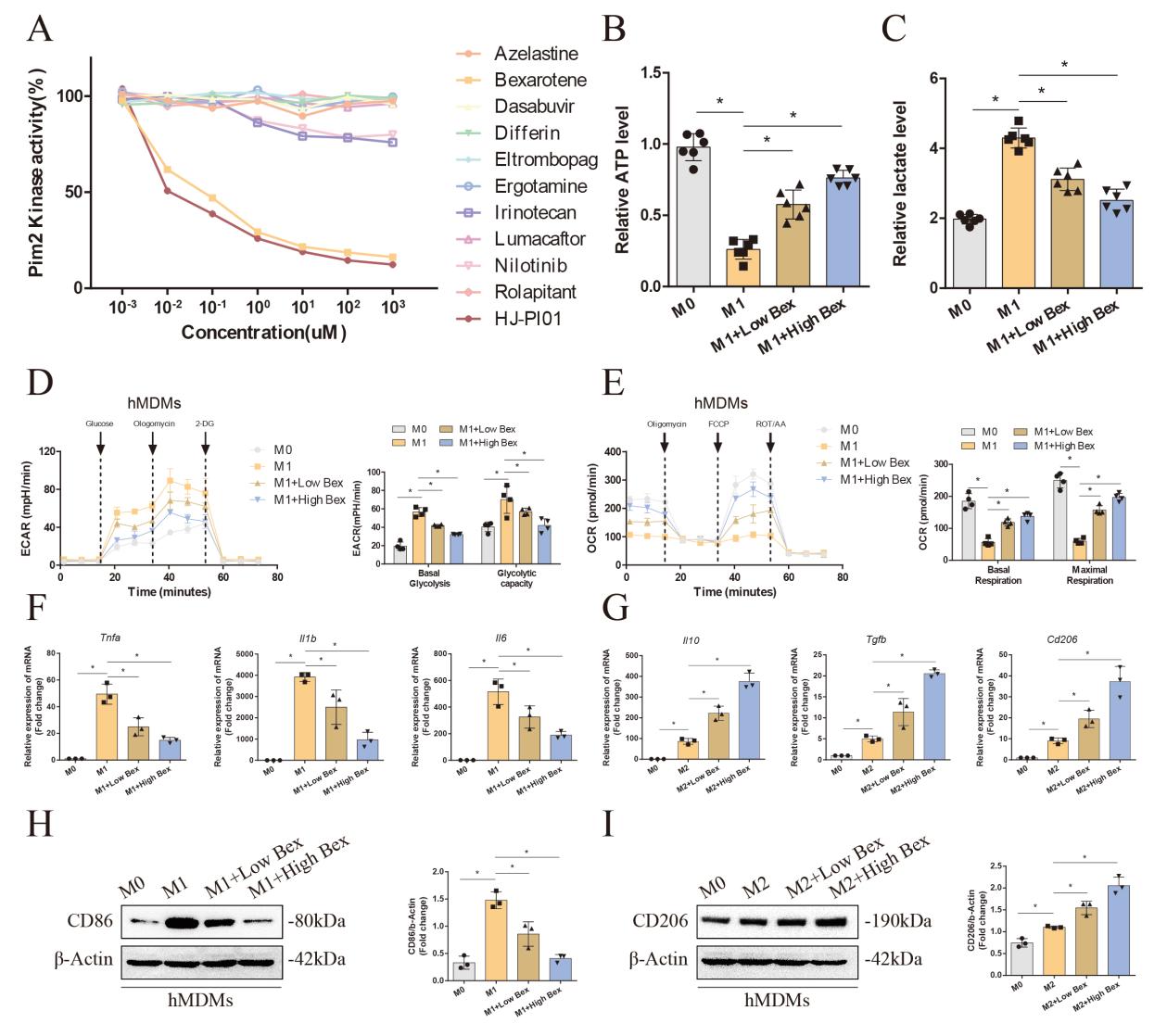


**Fig. S8. Bexarotene inhibits glycolysis and M1 polarization in macrophages by targeting Pim2**

A. Enzymatic activity of Pim2 after incubation with HJ-PI01, azelastine, bexarotene, dasabuvir, differin, eltrombopag, ergotamine, irinotecan, lumacaftor, nilotinib, or rolapitant (n=3). B-C. Relative levels of ATP and lactate in hMDMs treated with bexarotene(200 or 400 nM) (n=6). D-E. ECAR and OCR of hMDMs treated with bexarotene(200 or 400 nM) (n=4). F-I. The mRNA and protein expression levels of inflammatory factors in hMDMs treated with bexarotene(200 or 400 nM) after M1/M2 induction, as determined by RT‒qPCR, Western blotting (n=3).


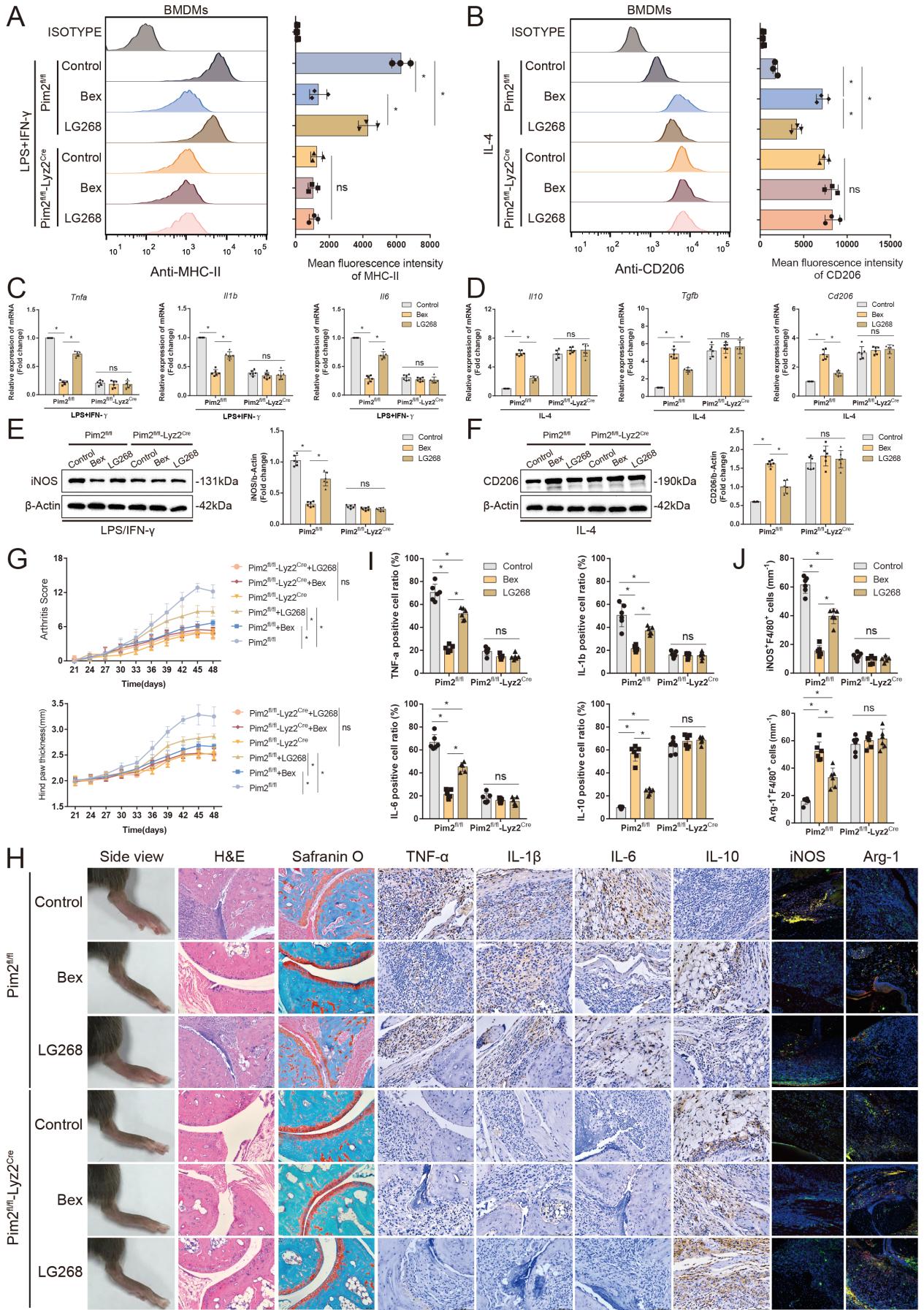


**Fig. S9. RXR activation is at least partially involved in the in vitro and in vivo anti-inflammatory effects of bexarotene.**

A-B. The MFI of MHC-II and CD206 in F4/80+ BMDMs treated with or without Bex(400 nM) and LG-268(500 nM) after M1/M2 induction , as detected by flow cytometry (n=6). C-F. The mRNA and protein expression levels of inflammatory factors in BMDMs treated with or without Bex(400 nM) and LG268(500 nM) after M1/M2 induction, as determined by RT‒qPCR, Western blotting (n=6). G. CIA scores and hind paw thickness of mice in each group (n=6). H. Macroscopic images, H&E, and Safranin O staining of the ankles from mice in each group (n=6). I. The expression of TNF-α, IL-1β, IL-6 and IL-10 in mice in each group, as detected by IHC (n=6). J. The expression of iNOS and Arg-1 in the synovial macrophages of mice in each group, as detected by immunofluorescence staining (F4/80, green; iNOS, red; Arg-1, red) (n=6).


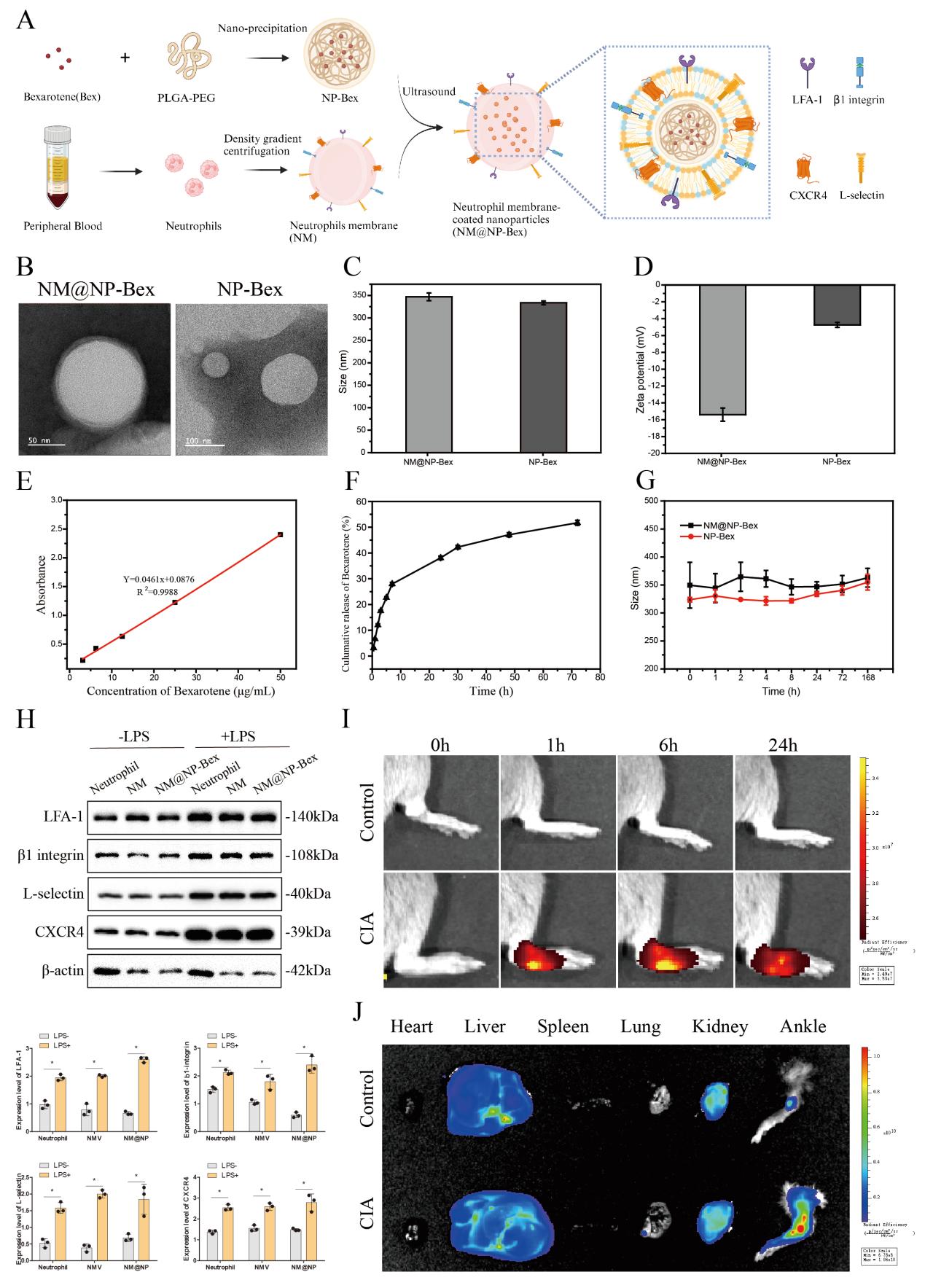


**Fig. S10. Characterization and Targeting of NM@NP-Bex**

A. Protocol for the synthesis of NM@NP-Bex. B. Representative transmission electron microscopy (TEM) images of NP-Bex and NM@NP-Bex. C. Average sizes of NP-Bex and NM@NP-Bex. D. Zeta potentials of NP-Bex and NM@NP-Bex. E. Standard curve for NP-Bex. F. In vitro release of bexarotene from NM@NP-Bex in PBS. G. Stability of NP-Bex and NM@NP-Bex as determined by DLS. H. The expression of LFA-1, β1-integrin, L-selectin, and CXCR4 on the neutrophil surface was detected by Western blotting. I. Real-time fluorescence imaging of CIA model mice after intravenous injection of Dil-labeled NM@NP-Bex. J. Ex vivo imaging of the organs at 24 hours after intravenous injection.


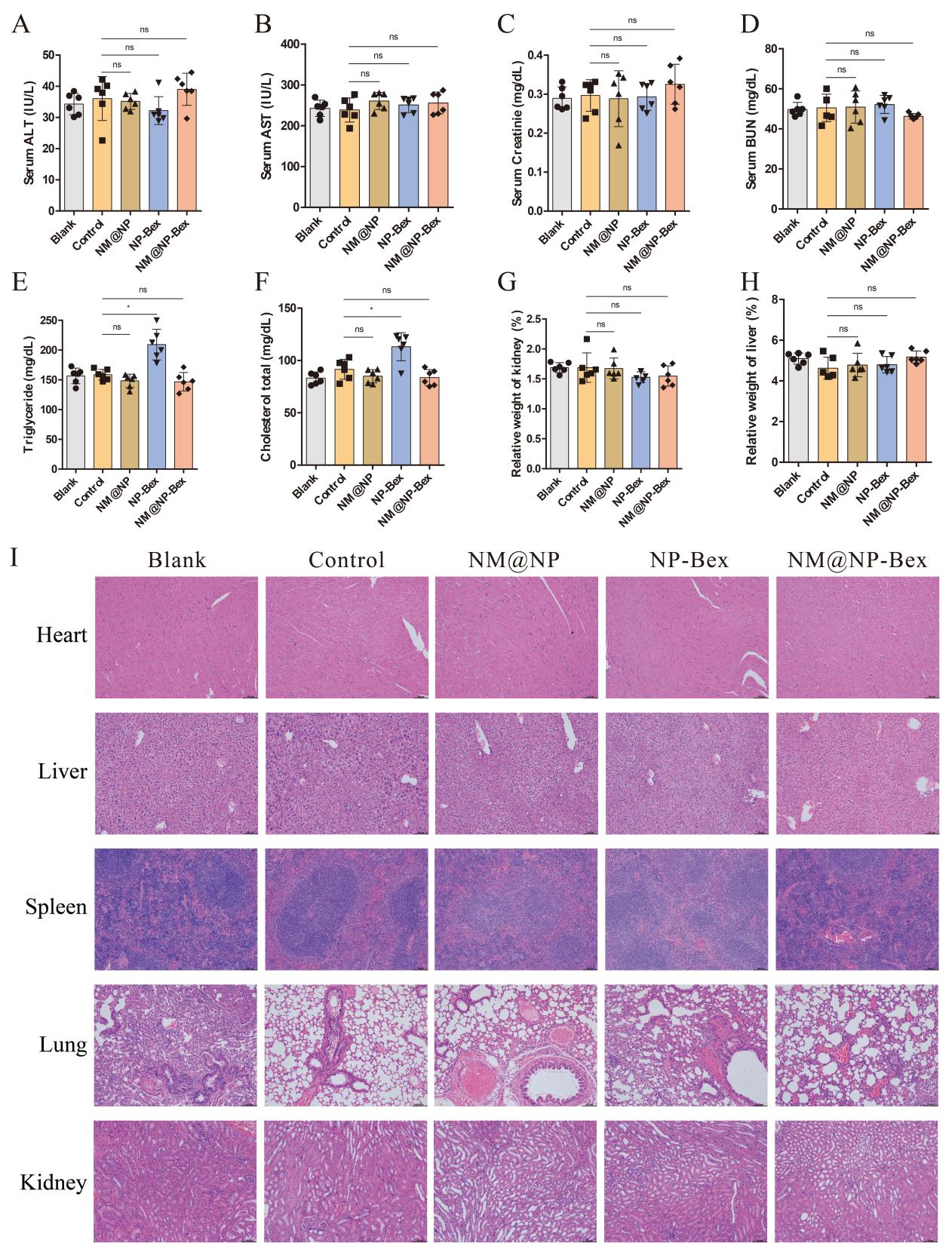


**Fig. S11. In vivo toxicity of NM@NP-Bex in CIA model mice**

1. F. Serum levels of ALT, AST, creatinine, BUN, triglyceride and cholesterol in CIA model mice after different treatments (n=6). G-H, Relative kidney and liver weights of CIA model mice after different treatments. I. H&E staining of major organs from mice after administration of different treatments (n=6).


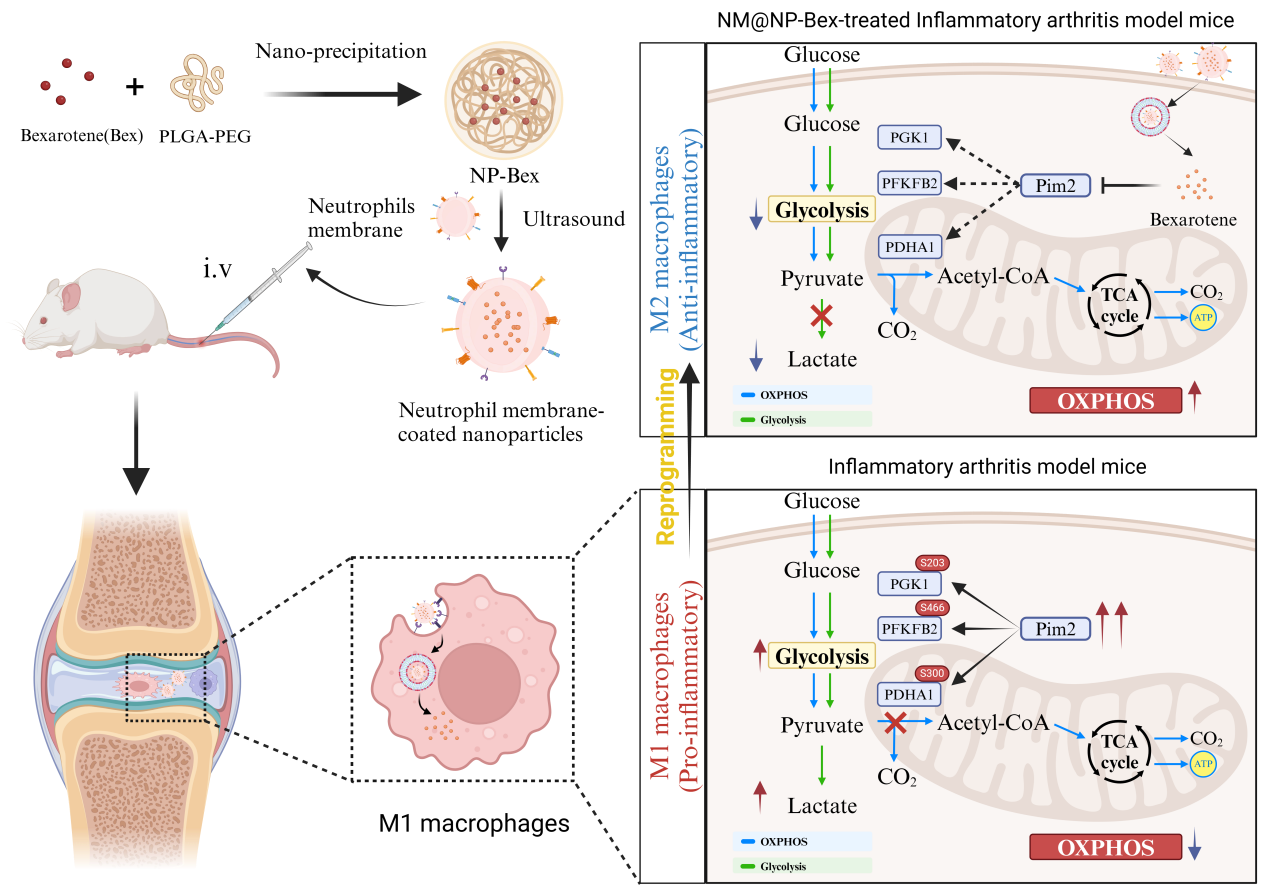


**Fig. S12. A schematic diagram of targeting macrophage polarization by inhibiting Pim2 alleviates inflammatory arthritis via aerobic glycolytic reprogramming.**

**
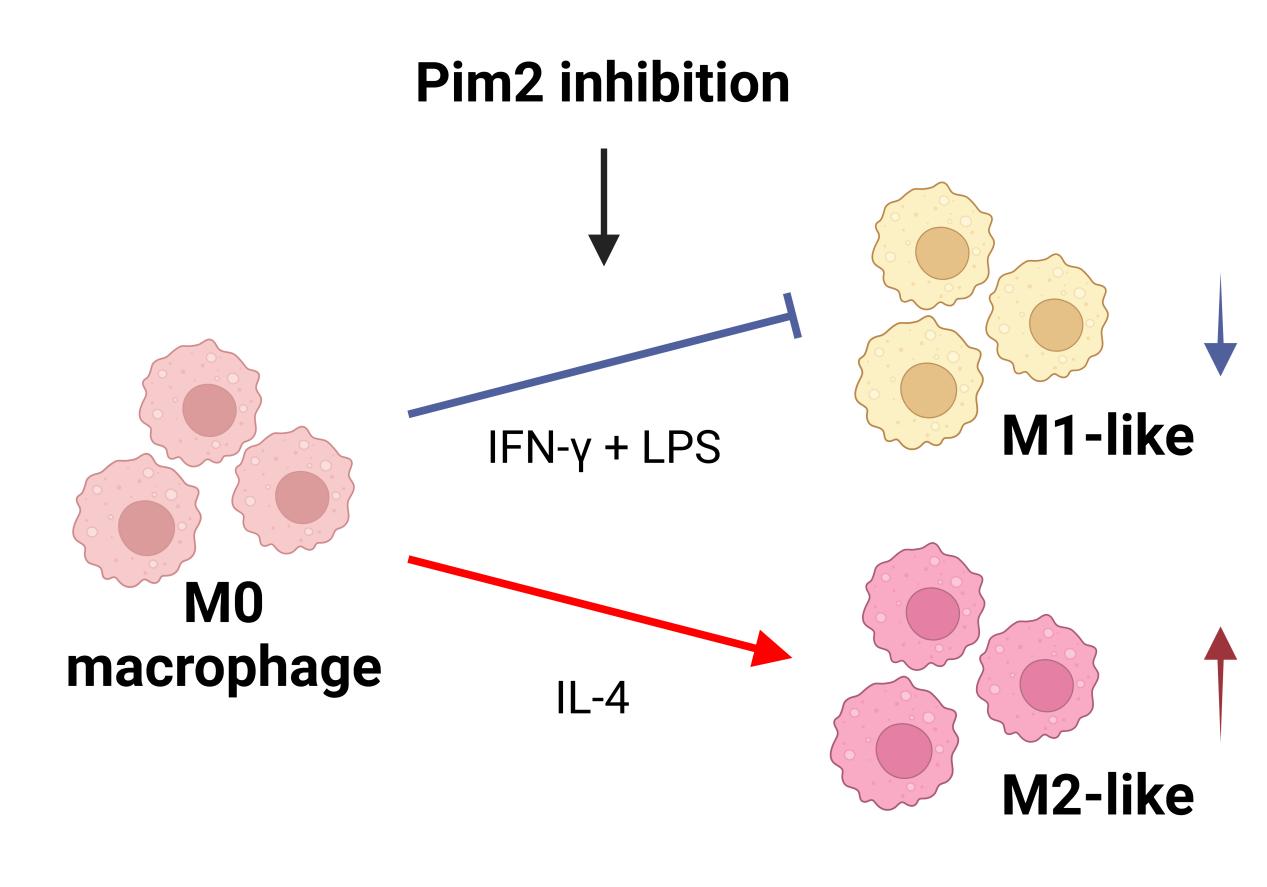
**

**Fig. S13. A schematic diagram showing the timming and effects of Pim2 inhibition on macrophage M1/M2 polarization**

**Supplementary Tables**

**Table S1: Binding pockets prediction for chain A of PIM2 main protease crystal (2IWI)**

| No.1 | Volume Å³ | Surface Å² | Drug Score | Simple Score |
| --- | --- | --- | --- | --- |
| 1 | 1391.62 | 1639.32 | 0.81 | 0.65 |
| 2 | 702.78 | 954.94 | 0.88 | 0.44 |
| 3 | 192.9 | 411.71 | 0.5 | 0.13 |
| 4 | 164.86 | 287.66 | 0.25 | 0.02 |
| 5 | 112.45 | 266.11 | 0.35 | 0 |
| 6 | 107.33 | 50.5 | 0.37 | 0 |
| 7 | 105.54 | 233.39 | 0.2 | 0 |

**Table S2: Binding pockets prediction for chain A of PIM2 main protease crystal(4X7Q)**

| No. | Volume Å³ | Surface Å² | Drug Score | Simple Score |
| --- | --- | --- | --- | --- |
| 1 | 868.54 | 1113.76 | 0.83 | 0.64 |
| 2 | 398.78 | 520.13 | 0.78 | 0.23 |
| 3 | 150.46 | 302.61 | 0.3 | 0 |
| 4 | 133.38 | 273.2 | 0.26 | 0.02 |
| 5 | 123.84 | 262.19 | 0.26 | 0 |
| 6 | 108.35 | 258.13 | 0.25 | 0 |
| 7 | 104.13 | 226.81 | 0.24 | 0 |
| 8 | 102.78 | 280.35 | 0.24 | 0.09 |

**Table S3: Chemical characteristics of the ten drugs as screened virtually against PIM2 main protease(2IWI and 4X7Q)**

| Molecules | 2D structure | Binding Affinity with 2IWI  (kcal/mol) | Binding Affinity with 4X7Q  (kcal/mol) | Formula | ZINC Number |
| --- | --- | --- | --- | --- | --- |
| Azelastine | 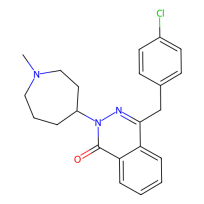 | -10.9 | -11.6 | C22H24ClN3O | ZINC000000897240 |
| Bexarotene | 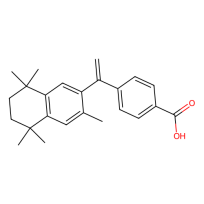 | -11.1 | -12.9 | C24H28O2 | ZINC000001539579 |
| Dasabuvir | 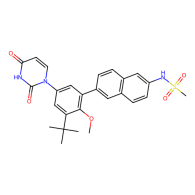 | -10.9 | -12 | C26H27N3O5S | ZINC000095616937 |
| Differin | 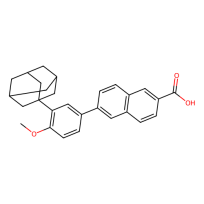 | -11.5 | -11.5 | C28H28O3 | ZINC000003784182 |
| Eltrombopag | 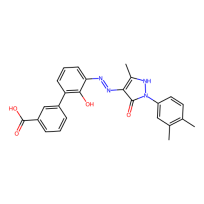 | -11.9 | -12.1 | C25H22N4O4 | ZINC11679756 |
| Ergotamine | 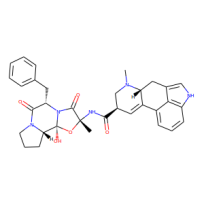 | -11.2 | -11.6 | C33H35N5O5 | ZINC52955754 |
| Irinotecan | 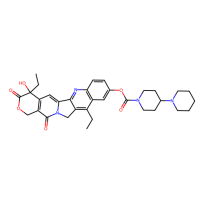 | -11.1 | -11.9 | C33H38N4O6 | ZINC000001612996 |
| Lumacaftor | 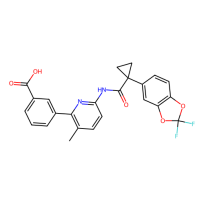 | -11 | -11.9 | C24H18F2N2O5 | ZINC000064033452 |
| Nilotinib | 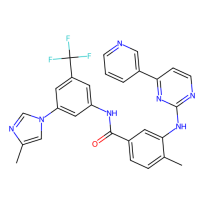 | -11.9 | -12.2 | C28H22F3N7O | ZINC000006716957 |
| Rolapitant | 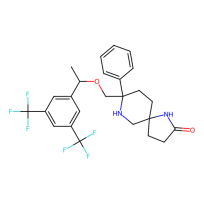 | -11.1 | -12.2 | C25H26F6N2O2 | ZINC000003816514 |

**Table S4. Characteristics of the OA and RA patients**

|  | OA | RA |
| --- | --- | --- |
| Number | 6 | 6 |
| Age，years(±SD) | 59.2 ±4.6 | 57.7 ±5.1 |
| Sex | Female | Female |
| Disease duration, years(±SD) | - | 13.5 ±5.2 |
| RF, IU/mL(±SD) | - | 148.2 ±46.1 |
| CRP, mg/mL(±SD) | 3.3 ±3.5 | 12.6 ±9.4 |
| ESR, mm/h(±SD) | 23.1 ±4.2 | 35.1 ±6.1 |
| Anti-CCP ab, U/mL(±SD) | - | 345.7 ±112.4 |

⚫ Data are expressed as mean ± standard deviation

⚫ CRP, C-reactive protein; ESR, erythrocyte sedimentation rate; Anti-CCP ab, Anti-Cycloguanidinopeptide (CCP) Antibody; ns, nonsense.

**Table S5. Sequences of siRNAs used for gene knockdown**

| **siRNA** | **Sequence (5’-3’)** | |
| --- | --- | --- |
| *NC* | Sense | UUCUCCGAACGUGUCACGUdTdT |
|  | Antisense | ACGUGACACGUUCGGAGAAdTdT |
| *PIM2 S1* | Sense | CCAGGAUCUCUUUGACUAUdTdT |
|  | Antisense | AUAGUCAAAGAGAUCCUGGdTdT |
| *PIM2 S2* | Sense | GCAUCCUCCUCUAUGACAUdTdT |
|  | Antisense | AUGUCAUAGAGGAGGAUGCdTdT |
| *PIM2 S3* | Sense | GCUAUGGAAAGUGGGUGCAdTdT |
|  | Antisense | UGCACCCACUUUCCAUAGCdTdT |

**Table S6. Primers used for qRT-PCR**

| **Gene** |  | **Primer sequence (5’-3’)** |
| --- | --- | --- |
| *β-Actin(Human)* | Forward | CAACCCCAAAAGAGCCAAACT |
|  | Reverse | CCTCGTAGAAGCTCCGACAGT |
| *PIM2(Human)* | Forward | CGAATCGCCGTGAAGAAAGC |
|  | Reverse | CTTGCAGGCGGAGTACACT |
| *IL-10(Human)* | Forward | AGCCAAGCGGCCTAAACTC |
|  | Reverse | TCACCACCATTGGTTAGTCCC |
| *TNF-α(Human)* | Forward | GCAAGGCCAATGAAACCTGTA |
|  | Reverse | TCCAAGTGGGATACGTGGTCA |
| *IL-6(Human)* | Forward | GAGGCCAAGCCCTGGTATG |
|  | Reverse | CGGGCCGATTGATCTCAGC |
| *IL-1β(Human)* | Forward | ACTCACCTCTTCAGAACGAATTG |
|  | Reverse | CCATCTTTGGAAGGTTCAGGTTG |
| *PGK1(Human)* | Forward | ATGATGGCTTATTACAGTGGCAA |
|  | Reverse | GTCGGAGATTCGTAGCTGGA |
| *PDHA1(Human)* | Forward | TGGTAGCATCCCGTAATTTTGC |
|  | Reverse | ATTCGGCGTACAGTCTGCATC |
| *PFKFB2(Human)* | Forward | TGGGCCTCCTACATGACCAA |
|  | Reverse | CAGTTGAGGTAGCGTGTTAGTTT |
| *β-Actin(Mouse)* | Forward | GTGACGTTGACATCCGTAAAGA |
|  | Reverse | GCCGGACTCATCGTACTCC |
| *PIM2(Mouse)* | Forward | TTCAGCGGGCTCAATATACGC |
|  | Reverse | CCAAGTCGGTATTCGGCCTC |
| *IL-10(Mouse)* | Forward | CTTACTGACTGGCATGAGGATCA |
|  | Reverse | GCAGCTCTAGGAGCATGTGG |
| *TNF-α(Mouse)* | Forward | CAGGCGGTGCCTATGTCTC |
|  | Reverse | CGATCACCCCGAAGTTCAGTAG |
| *IL-6(Mouse)* | Forward | CTGCAAGAGACTTCCATCCAG |
|  | Reverse | AGTGGTATAGACAGGTCTGTTGG |
| *IL-1β (Mouse)* | Forward | GAAATGCCACCTTTTGACAGTG |
|  | Reverse | TGGATGCTCTCATCAGGACAG |

**Table S7. Anibodies used for Western Blotting**

| **Antibody** | **Species** | **Source** | **Catalog** | **Dilution** | **Application** |
| --- | --- | --- | --- | --- | --- |
| *Anti-Flag* | *R* | Cell signaling technology | 14793 | WB: 1:1000  IP: 1:50 | WB, IP |
| *Anti-HA* | *R* | Cell signaling technology | 3724 | WB: 1:1000  IP: 1:50 | WB, IP |
| *Anti-iNOS* | *R* | Abcam | ab178945 | WB: 1:1000  IF: 1:50 | WB, IF |
| *Anti-CD86* | *R* | Abcam | ab239075 | 1:1000 | WB |
| *Anti-CD206* | *R* | Abcam | ab64693 | 1:1000 | WB |
| *Anti-Arg-1* | *R* | Cell signaling technology | 93668 | WB: 1:1000  IF: 1:50 | WB, IF |
| *Anti-β-Actin* | *M* | Cell signaling technology | 4967 | 1:2000 | WB |
| *Anti-PIM2* | *R* | Abcam | ab129057 | WB: 1:1000  IP: 1:100  IF: 1:100 | WB, IP, IF |
| *Anti-IL-10* | *R* | Abcam | ab133575 | 1:50 | IHC |
| *Anti-TNF-α* | *R* | Abcam | ab183218 | 1:50 | IHC |
| *Anti-IL-6* | *R* | Abcam | ab233706 | 1:50 | IHC |
| *Anti-IL-1β* | *R* | Abcam | ab315084 | 1:50 | IHC |
| *Anti-PGK1* | *R* | Abcam | ab199438 | WB: 1:1000  IP: 1:100  IF: 1:100 | WB, IP, IF |
| *Anti-PDHA1* | *R* | Abcam | ab168379 | WB: 1:1000  IP: 1:100  IF: 1:100 | WB, IP, IF |
| *Anti-PFKFB2* | *R* | Abcam | ab241506 | WB: 1:2000  IP: 2-10 µg/mg | WB, IP |
| *Anti-PFKFB2* | *R* | Abcam | ab234865 | 1:100 | IF |
| *PE Mouse Anti-Human HLA-DR* | *M* | BD Pharmingen | TU36 | 5ul/test | FC |
| *PE Mouse Anti-Human CD86(2331 (FUN-1)* | *M* | BD Pharmingen | 555658 | 5ul/test | FC |
| *BV421 Mouse Anti-Human CD206(19.2)* | *M* | BD Pharmingen | 566281 | 5ul/test | FC |
| *BV421 Mouse Anti-Human CD163(GHI/61)* | *M* | BD Pharmingen | 566277 | 5ul/test | FC |
| *FITC Rat Anti-Mouse I-A/I-E(2G9)抗体* | *R* | BD Pharmingen | 562009 | 5ul/test | FC |
| *Alexa Fluor 647 Rat Anti-Mouse CD206(MR5D3)* | *R* | BD Pharmingen | 565250 | 5ul/test | FC |
| *FITC Rat Anti-Mouse CD86(GL1)抗体* | *R* | BD Pharmingen | 561962 | 5ul/test | FC |
| *Alexa Fluor 647 Mouse Anti-Rat IL-10(A5-4)* | *R* | BD Pharmingen | 562165 | 5ul/test | FC |
| *Alexa Fluor 488-conjugated goat anti-mouse IgG secondary antibody* | *G* | Invitrogen | A- 11001 | 1:500 | IF |
| *Alexa Fluor® 488-conjugated goat anti-rabbit IgG secondary antibody* | *G* | Invitrogen | A-11008 | 1:500 | IF |
| *Alexa Fluor™ 555-conjugated goat anti-mouse IgG secondary antibody* | *G* | Invitrogen | A-21422 | 1:500 | IF |
| *Alexa Fluor 555-conjugated goat anti-rabbit IgG secondary antibody* | *G* | Invitrogen | A- 21428 | 1:500 | IF |
| L-selectin | R | R&D systems | MAB1534 | 1:1000 | WB |
| CXCR4 | R | Abcam | ab124824 | 1:1000 | WB |
| LFA-1 | R | Abcam | ab13219 | 1:1000 | WB |
| β1 integrin | R | Abcam | EPR16895 | 1:1000 | WB |
