# Supplementary figures and images for: Targeting macrophage polarization by inhibiting Pim2 alleviates inflammatory arthritis via metabolic reprogramming
Source: Cell Mol Immunol. 2025 Feb 26;22(4):418–36. doi: 10.1038/s41423-025-01268-9 (PMC11955556; doi:10.1038/s41423-025-01268-9)

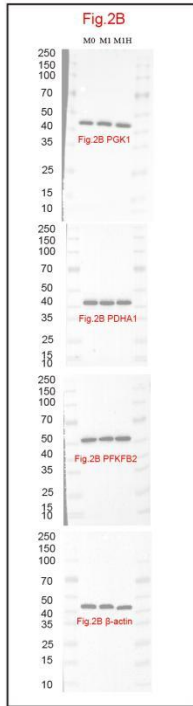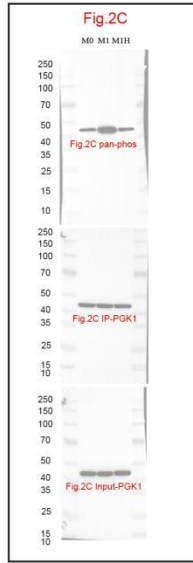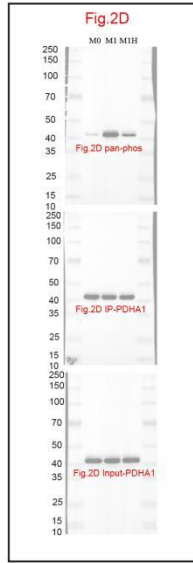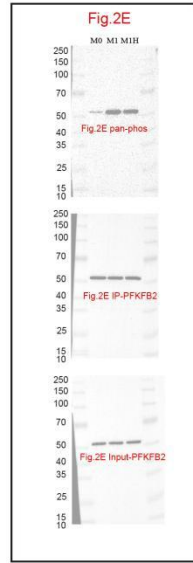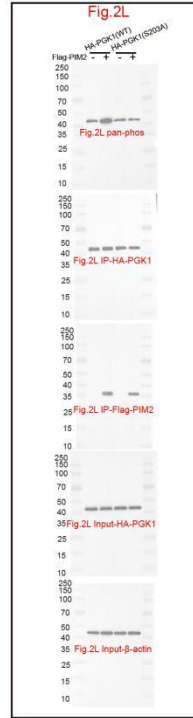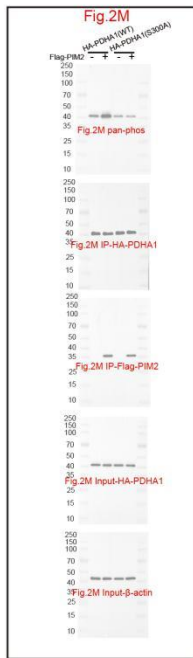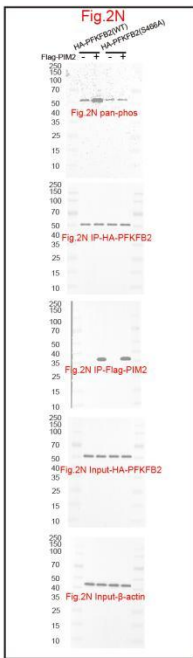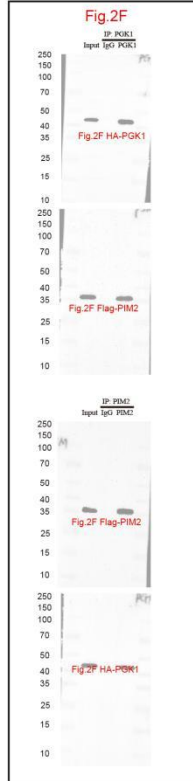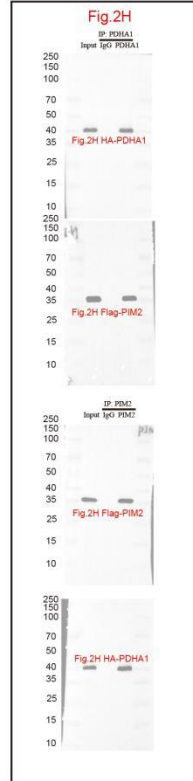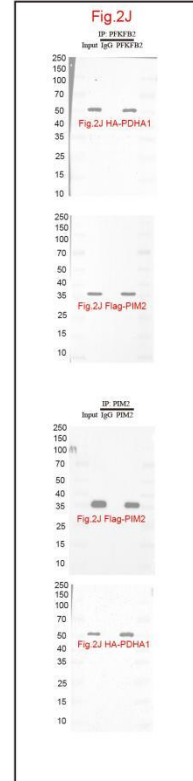

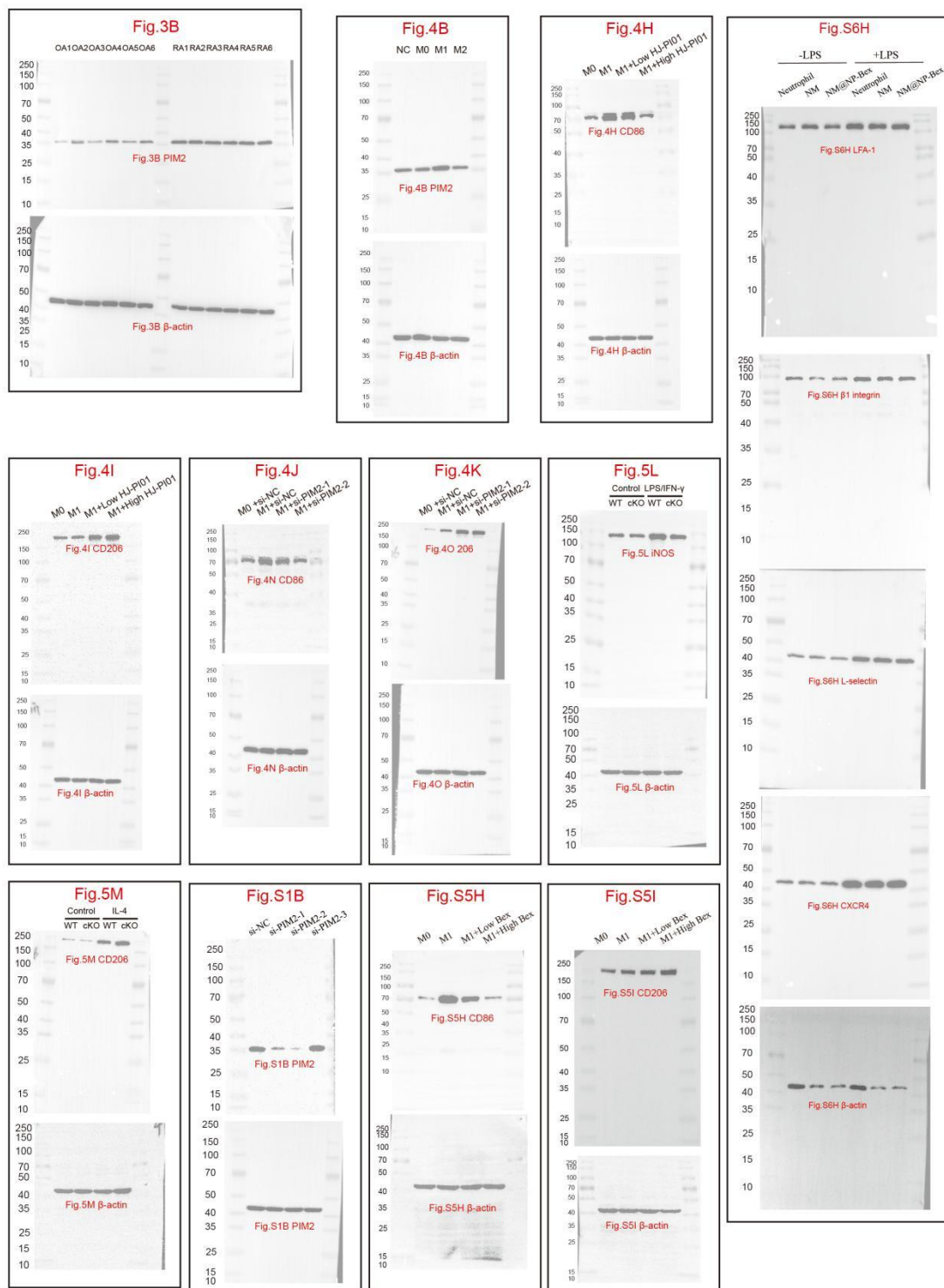

Fig. 2A

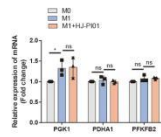

Fig. 3C

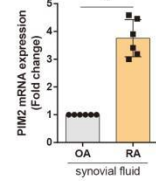

Fig. 4A

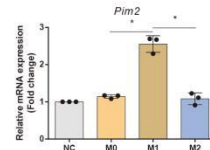

Fig. 4F

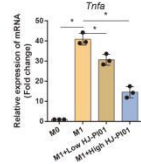

Fig. 4G

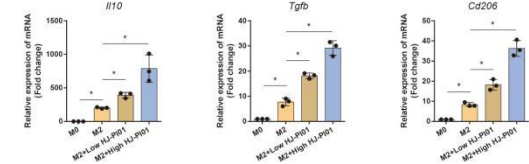

Fig. 4L

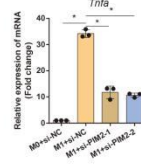

Fig. 4M

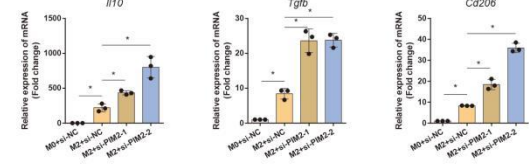

Fig. 5L

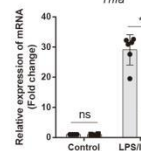

Fig. 5M

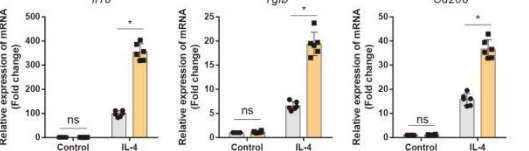

Fig. S1B

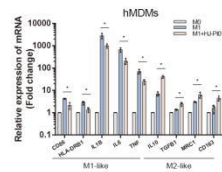

Fig. S1C

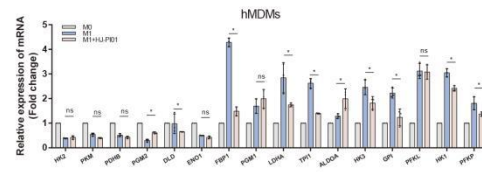

Fig. S2A

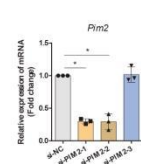

Fig. S4A

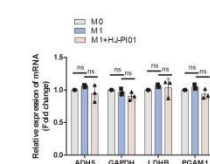

Fig. S5B

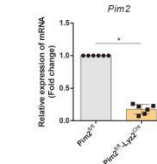

Fig. S6F

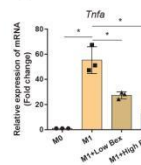

Fig. S6G

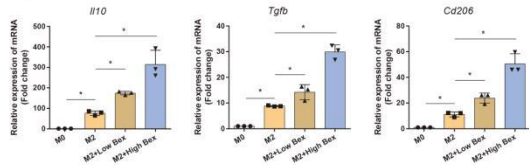

Supplement: Supplementary file 2 — unprocessed original images [file 41423_2025_1268_MOESM2_ESM.pdf]
